# Climate change and the global redistribution of biodiversity: substantial variation in empirical support for expected range shifts

**DOI:** 10.1186/s13750-023-00296-0

**Published:** 2023-04-11

**Authors:** Madeleine A. Rubenstein, Sarah R. Weiskopf, Romain Bertrand, Shawn L. Carter, Lise Comte, Mitchell J. Eaton, Ciara G. Johnson, Jonathan Lenoir, Abigail J. Lynch, Brian W. Miller, Toni Lyn Morelli, Mari Angel Rodriguez, Adam Terando, Laura M. Thompson

**Affiliations:** 1 U.S. Geological Survey (USGS), National Climate Adaptation Science Center, Reston, USA; 2 Université de Toulouse 3, Toulouse, France; 3 School of Biological Sciences, Illinois State University, Normal, USA; 4 Southeast Climate Adaptation Science Center, USGS, Raleigh, USA; 5 Université de Picardie Jules Verne, Amiens, France; 6 North Central Climate Adaptation Science Center, USGS, Boulder, USA; 7 Northeast Climate Adaptation Science Center, USGS, Amherst, USA; 8 Department of Environmental Science & Policy, George Mason University, Fairfax, USA

**Keywords:** Global change, Distribution shift, Species redistribution, Latitude, Elevation, Depth, Warming, Vulnerability

## Abstract

**Background:**

Among the most widely predicted climate change-related impacts to biodiversity are geographic range shifts, whereby species shift their spatial distribution to track their climate niches. A series of commonly articulated hypotheses have emerged in the scientific literature suggesting species are expected to shift their distributions to higher latitudes, greater elevations, and deeper depths in response to rising temperatures associated with climate change. Yet, many species are not demonstrating range shifts consistent with these expectations. Here, we evaluate the impact of anthropogenic climate change (specifically, changes in temperature and precipitation) on species’ ranges, and assess whether expected range shifts are supported by the body of empirical evidence.

**Methods:**

We conducted a Systematic Review, searching online databases and search engines in English. Studies were screened in a two-stage process (title/abstract review, followed by full-text review) to evaluate whether they met a list of eligibility criteria. Data coding, extraction, and study validity assessment was completed by a team of trained reviewers and each entry was validated by at least one secondary reviewer. We used logistic regression models to assess whether the direction of shift supported common range-shift expectations (i.e., shifts to higher latitudes and elevations, and deeper depths). We also estimated the magnitude of shifts for the subset of available range-shift data expressed in distance per time (i.e., km/decade). We accounted for methodological attributes at the study level as potential sources of variation. This allowed us to answer two questions: (1) are most species shifting in the direction we expect (i.e., each observation is assessed as support/fail to support our expectation); and (2) what is the average speed of range shifts?

**Review findings:**

We found that less than half of all range-shift observations (46.60%) documented shifts towards higher latitudes, higher elevations, and greater marine depths, demonstrating significant variation in the empirical evidence for general range shift expectations. For the subset of studies looking at range shift rates, we found that species demonstrated significant average shifts towards higher latitudes (average = 11.8 km/dec) and higher elevations (average = 9 m/dec), although we failed to find significant evidence for shifts to greater marine depths. We found that methodological factors in individual range-shift studies had a significant impact on the reported direction and magnitude of shifts. Finally, we identified important variation across dimensions of range shifts (e.g., greater support for latitude and elevation shifts than depth), parameters (e.g., leading edge shifts faster than trailing edge for latitude), and taxonomic groups (e.g., faster latitudinal shifts for insects than plants).

**Conclusions:**

Despite growing evidence that species are shifting their ranges in response to climate change, substantial variation exists in the extent to which definitively empirical observations confirm these expectations. Even though on average, rates of shift show significant movement to higher elevations and latitudes for many taxa, most species are not shifting in expected directions. Variation across dimensions and parameters of range shifts, as well as differences across taxonomic groups and variation driven by methodological factors, should be considered when assessing overall confidence in range-shift hypotheses. In order for managers to effectively plan for species redistribution, we need to better account for and predict which species will shift and by how much. The dataset produced for this analysis can be used for future research to explore additional hypotheses to better understand species range shifts.

**Supplementary Information:**

The online version contains supplementary material available at 10.1186/s13750-023-00296-0.

## Background

Contemporary climate change represents one of the foremost drivers of ecological change, yet its current and future impacts on species, communities, and distributions remain uncertain. Such uncertainty impedes effective planning and decision making for conservation and natural resource management (e.g., spatial conservation planning, corridor designs, endangered species listings). This uncertainty is driven in part by large variability in biological responses to climate change [1]. Despite a range of commonly-held hypotheses supported by ecological theory, many species are responding in counter-intuitive ways [2–4]. Among the most significant and widely discussed of these expectations are shifts in species’ spatial distributions (i.e., range shifts). Range shifts have the potential to reshape ecological communities, alter ecosystem functions and the provision of ecosystem services, impact human health and well-being, and even have feedback effects on the climate system [5]. Understanding how species are shifting as a function of climate change is important for effectively managing species and habitats.

Climate-change-related range shifts (hereafter “range shifts”) are well documented [6–9] and relatively well studied [10–12]. Over the past two decades, as the research community has conducted an increasing number of studies devoted to documenting range shifts [13], a series of broad hypotheses have emerged. Generally, these hypotheses predict that species will track their climatic niches along spatial gradients [8, 14–16]. Niche tracking is easier to predict for temperature (i.e., isotherm tracking) than precipitation (i.e., isohyet tracking), as temperatures generally decrease with increasing terrestrial latitude and elevation, and with increasing depth in freshwater and marine environments [8, 17]. In particular, there are three prominent directional outcomes in terms of species range shifts expected from the isotherm-tracking hypothesis [18]: (i) poleward to higher latitudes; (ii) upslope to higher elevations (for terrestrial and freshwater species); and (iii) to greater depths (for freshwater and marine species) [6, 7]. In addition, the leading edge (e.g., the poleward or upslope edge of species’ ranges) is generally expected to shift more rapidly in response to rises in temperature than the trailing edge (e.g., equatorial or downslope edge) or the center of species’ ranges [8, 19, 20]. Although there are no clear directional outcomes for species range shifts in response to changing precipitation patterns, generally species are expected to shift their ranges to maintain optimal precipitation envelopes (i.e., isohyet tracking [14, 21, 22]). The expected directional outcomes for temperature have become so widespread that many reports generally assume that species should shift poleward, to higher elevations, and to greater depths without considering local isotherms or other abiotic dimensions of the climatic niche space, limiting our ability to infer mechanisms for non-conforming responses (but see [1, 2, 8, 23]).

Previous reviews have demonstrated general support across taxonomic groups and regions for expected latitudinal, elevation, and depth shifts [7–9]; yet, substantial variation across species, systems, and regions has also been documented [1, 9, 24]. Numerous studies have found that many species, populations, and taxonomic groups have not demonstrated the expected shifts, or have even displayed counterintuitive shifts (e.g., equatorial or downslope movement) [1, 25, 26]. Moreover, previous reviews have been hampered by a number of limitations, including: (i) combination of or unspecified range-shift parameters (e.g., leading edge, trailing edge, and center-of-range) [8, 27]; (ii) omission of studies measuring changes in abundance [28]; and (iii) exclusion of single-species studies in an attempt to avoid publication bias [9]. Given the ubiquity of climate change impacts, and the important role of climate in determining species’ ranges, the substantial body of evidence that does not conform to hypothesized range shift expectations deserves greater investigation. An updated assessment and repository of range-shift studies that highlight the evidence for and against hypothesized range shift expectations are needed to clarify sources of variation in the empirical data. Indeed, a recent survey by the U.S. Association of Fish and Wildlife Agencies identified research on range shifts as a priority need for coastal and marine natural resource managers, indicating the benefits of additional synthesis for improving wildlife and habitat management [29]. Given this identified gap, the research team, composed of U.S. government employees and university researchers, developed and conducted this review.

## Objective of the review

The objective of this review is to provide a robust assessment of evidence for a series of general range-shift expectations in response to climate change for plants and animals across terrestrial, freshwater, and marine ecosystems. These expectations, while based on isotherm- and isohyet-specific tracking hypotheses, are broadly formulated: species are generally expected to shift their ranges to higher latitudes, higher elevations, and greater depths to maintain their temperature niche, and to shift in various ways to maintain their precipitation niche (e.g., if precipitation in a species’ range decreases, we expect the species to move to areas with higher precipitation, as assessed by the authors of the original studies). Following the PECO (Population, Exposure, Comparator, Outcome) question framework (Table 1) [30, 31], we identified four key elements of our study that structured and guided our literature search, including:

(i) population of interest—animal and plant species
(ii) exposure—climate change variables (temperature and precipitation)
(iii) comparator—baseline temperature and/or precipitation conditions during the historical period
(iv) outcome—measures of species range shift in spatial/geographic distribution

**Table 1:** Study eligibility criteria

PECO component	Include	Exclude
Population: Species of animals or plants	Articles that report on animals and plants, with results that are species- or subspecies specific	Articles that report on organisms not classified as animals or plants, or include results that are presented for broader taxonomic groups (genus, family, etc.) or on a community-or assemblage-level
Exposure: Climate change variables (temperature and precipitation)	Articles that study responses to contemporary changes in temperature or precipitation due to modern, anthropogenic climate change	Articles that are based on paleoclimatic data, future climate change scenarios, temporary climate changes (e.g., ENSO, marine heatwaves) or seasonal climate variation, as well as articles that document range shifts in response to non-temperature or non-precipitation related climate variables (e.g., sea level rise, ocean acidification)
Comparator: Baseline temperature and/or precipitation conditions during the historical period	Articles that use empirical observations to describe changes in species distribution and/or range limits by comparing the contemporary observation(s) with at least one observation of the same type in the past	Articles that only predict future species’ range/distribution shifts based on simulations or projections, and articles that only use observations from a single point in time or those that do not specify the temporal scale
Outcome: Shift in spatial/geographic distribution	Articles that report or attempt to document a change in species’ range or spatial distribution (i.e., boundary level shifts), or spatial shifts in abundance (i.e., distribution within a range)	Articles that use proxy measurements to document changes in a species range (e.g., measuring species’ thermal tolerance or limits, measuring changes in adult to juvenile ratios)

We assessed the overall direction and magnitude of species range shifts and evaluated variation across taxonomic groups. Analyzing direction of shift allowed us to also consider studies that reported range shifts qualitatively rather than quantitatively (e.g., study reported that a species moved north during the study period, but did not provide the shift value in a measurable unit), which has not been done systematically before. This allowed us to answer two questions: (1) are most species shifting in the direction we expect (i.e., each observation is assessed as support/fail to support our expectation); and (2) what is the average speed and direction of range shifts? We improved upon previous studies by analyzing range-shift parameters separately, as well as by incorporating single-species studies and studies which measured range shifts through changes in local abundance. We assessed the degree to which documented observations support hypothesized range-shift expectations. We collected documented examples of climate-change-driven range shifts globally, including studies appropriately designed to document shifts but which failed to detect them, and assessed the body of evidence in terms of both magnitude (i.e., average km/decade shifts) and direction of species range shifts (i.e., consistent or inconsistent with hypothesized expectations).

## Methods

A full accounting of our systematic review methods is available in [32].We added slight updates and additional details to the data synthesis and presentation section to track the final analyses (e.g., we excluded longitudinal range shift studies from the final analysis given the limited number of observations and difficulty of linking with temperature-related range shift hypotheses). Below, we provide an updated description of our systematic review process.

### Searching for articles

#### Online databases

**Fig. 1:**
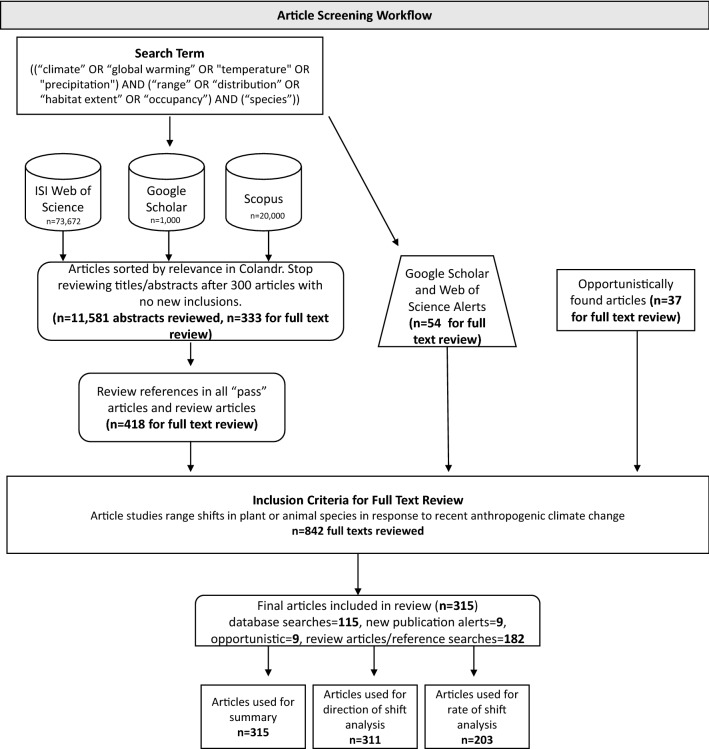
Systematic Review workflow diagram

We conducted the review using the advanced search tool in two online databases: *Web of Science* and *Scopus* (Fig. 1). For *Web of Scienc*e, we used the Science Citation Index Expanded Science collection within the Core Collection Database. We selected the ‘all years (1985 to 2019)’ timespan.

#### Online search engines

We then conducted a follow-up search in the online search engine *Google Scholar* (Fig. 1), and reviewed 1000 articles (the maximum retrievable; [36]); only articles not previously identified were included. We used the advanced search options and entered our search terms in the “with all of the words” field. Similar to the *Web of Science* and *Scopus* searches, our *Google Scholar* searches were not constrained by date, year, or author. All searches were conducted in English, since this is the primary language of the reviewers. We acknowledge a clear bias by not incorporating the non-English-language science that could impact the spatial representativeness of species redistribution (see Fig. 3b; [37–39]), and that the conclusions of our review should be interpreted in light of this gap. Although we did not search for any non-English articles, we did include them if they came up during the search (i.e., through the snowball method described below).

#### Search terms

We established a broad set of search terms based on our knowledge of climate change and ecological literature (Fig. 1) and conducted preliminary searches with different combinations of those terms. For each combination, we compared the total number of articles retrieved and the number of articles whose title and abstract were judged to be relevant based on our eligibility criteria in the first 500 returns. We selected our final search terms (Additional File 1) based on the combination of terms that returned the most relevant articles in the first 500 reviewed. We tested the comprehensiveness of the search terms by compiling a list of all articles included in a recent meta-analysis [7], as well as a few relevant articles chosen based on the author team’s previous knowledge of the literature (35 articles total; see Additional File 1 for complete list) and determining how many of them were successfully returned by the *Web of Science*, *Scopus*, and/or *Google Scholar* search results. All articles were returned in our search results.

#### Additional search methods

We used the snowball method [40], whereby we reviewed the cited literature within each article that passed title/abstract review to determine if any of those additional articles should have been included. We also screened opportunistically retrieved articles. These are articles that we came across outside of the formal search process, such as those sent to us by colleagues or found by chance in an unrelated capacity. We recorded the citation information of any opportunistically retrieved articles and reviewed this list for any potentially relevant articles not already identified through the *Web of Science*, *Scopus*, *Google Scholar*, or snowball search methodologies.

After we completed our initial review, we set up alerts for new articles that matched our search terms in *Google Scholar* and *Web of Science*: this allowed us to capture any newly published articles that may have come out after our search but before publication of this systematic review. Alerts were checked on a regular basis by a trained reviewer, and the articles listed in the alert were subject to title/abstract review. We did not include any new articles identified via alerts or opportunistically reviewed articles after June 2021.

#### Other considerations

We did not incorporate data from review articles or previous meta-analyses (e.g., [6, 9, 41]) directly into our review; instead, we used review articles and meta-analyses to identify original articles that may have contained relevant data. We flagged review articles and meta-analyses as “review” to be inspected after the preliminary title and abstract review. Each review article and its citations were read to identify the relevant primary source; we then evaluated the primary source and included it as appropriate based on a title/abstract review.

Our methodology is challenged with regards to reproducibility, given that our exact literature search in *Google Scholar* may not produce identical results in the future. Search results from these sources vary as a function of date and location. To address this, we maintained a spreadsheet of all search results and used *Colandr* to sort articles by relevance, which improves the transparency of this process.

#### Article screening and study eligibility criteria

We uploaded our search results to *Colandr*, a machine learning tool that iteratively sorts articles based on relevance as defined by the user (i.e., it continually learns to rank articles based on which articles are included and excluded) [33]. This process allowed us to review the most relevant articles based on our inclusion criteria (Table 1; see section below on eligibility criteria). Given the particularly broad scope of this review (i.e., global range shifts across all animals and plants from marine, freshwater, and terrestrial ecosystems), the number of retrieved articles exceeded our capacity to complete an exhaustive review of every article. To balance specificity with comprehensiveness, we constructed an accumulation curve to determine when to stop reviewing article abstracts following the method outlined in [34]. Using the *Colandr* relevance ranking function, highly relevant articles appeared earlier in the search. We reviewed abstracts 100 at a time, recording the number of included vs. excluded articles to create an accumulation curve (articles passing title/abstract review per 100 titles); once we reached the asymptote of the accumulation curve (defined by no new successful articles out of 300 titles reviewed), we stopped searching the database results. To validate our stopping criteria, we selected a random subset of 300 unreviewed articles for title and abstract review, and evaluated whether their inclusion substantially altered the findings of our meta-analysis (similar to the displacement method described in [35]). During this validation step in our review, we found only two additional articles that met our study inclusion criteria. We found that inclusion of these two articles did not change our overall results, and we therefore considered our search sufficient (Additional File 5: Table S1, see [35] for additional assessments of a similar search stopping criteria).

#### Screening process

We used a two-stage screening process to determine eligibility: (1) title/abstract review and (2) full text review. Internal reviewers (among our co-authors) independently reviewed the first 500 articles in stage 1 (a little over 4% of the total abstracts screened); decisions (e.g., eligible/ineligible) were compared and discrepancies discussed to ensure that eligibility criteria (Table 1) were being consistently applied. After the first 500 articles, we reviewed individual articles independently. In stage 2, any article that passed full text review was checked by a second author. Any questions about whether an article met screening criteria were discussed among co-authors.

During the first stage of the screening process, the reviewer read titles to determine relevance. For example, a relevant title might be one that contains one or more of our keywords, but not those that are clearly from another subject or field. If the title was determined to be relevant, the reviewer read the abstract and checked that the article contained the necessary components for our study (Table 1). If the abstract indicated that the study may meet the required components or that eligibility was unclear, the article was passed on to full-text review.

During the second stage, reviewers read articles in their entirety and verified that the article met all eligibility criteria. Any uncertainties about a paper were discussed with at least one additional co-author to resolve how to move forward. Reviewers that had authored an article under consideration were recused from decisions regarding the eligibility of the article. A full list of articles excluded from the first stage of title/abstract review and those excluded during the second stage at full-text review are available as a Additional File 2, including documented reasons for exclusion.

#### Eligibility criteria

To determine eligibility for inclusion in the review, we established eligibility criteria to align with PECO elements of our review, based on a priori familiarity with climate change research and the scope of our research objectives (e.g., [6, 8, 9, 41]), which were refined after preliminary scoping of the available literature (Table 1). Because the objective of our study was to assess empirical evidence for climate change-related range shifts, we only accepted articles that documented or attempted to document distributional shifts based on empirical observations, and not those that exclusively described projected future changes based on model predictions or simulations. Our review was focused on species-level range shifts, so that we could examine the variability of responses at this scale. Consequently, we only accepted articles reporting results at the species level or finer taxonomic resolutions (e.g., subspecies level). For articles that failed to report results at a species or subspecies level, but which appeared to have used underlying species-specific data, we contacted the corresponding author for data. Additionally, we only included articles reporting observed range shifts in animals and plants (excluding fungi), since these are the most well-represented taxonomic groups in the scientific literature and are likely to be of greater interest to natural resource managers. Since we are interested in understanding spatial changes in response to recent anthropogenic climate change, we did not include articles that were related to paleoclimatic conditions or temporary or seasonal climate variations, such as the El Niño-Southern Oscillation (ENSO). Although we did not impose a minimum study length for article inclusion in the database, we did impose additional criteria for inclusion in the data analysis portion of our review as required by analytical constraints (see *Data synthesis & Presentation* section). Our range-shift expectations are specific to trends in temperature and precipitation, so only studies that considered bioclimatic variables related to either temperature or precipitation were eligible (e.g., we did not include studies focused on range shifts related to sea level rise).

### Study validity assessment

All eligible studies were critically appraised for risk of bias and coded based on their methodology and data quality [8, 9]. Rather than excluding studies using certain methodologies, we included methodological metrics as covariates in our analyses. This enabled us to account for differences in methodology among studies and estimate the size and direction of bias associated with different study methods [42]. We categorized studies according to five methodological factors that may influence or bias study outcomes [9, 42]. These factor categories included:

- Number of taxa for which range-shift observations were recorded in the study (referred to as *ntax*), indicating potential positive publication bias [6, 7]: although some previous meta-analyses of range shifts have omitted single-species studies, we determined that accounting for this as a methodological approach could effectively allow for the inclusion of these data [9]. For our analyses, we categorized *ntax* into a binary factor variable of one or more than one species;
- Monitoring frequency (referred to as *sample*): regular (i.e., authors sampled species ranges continuously throughout the study period at specified time intervals) vs. irregular (i.e., authors sampled species ranges periodically throughout the study period at irregular time intervals);
- Underlying nature of the data, indicating whether abundance or occurrence data were collected (referred to as *obsvt*);
- Sampling design (referred to as *resurvey*), indicating whether the data were drawn from a balanced resurvey (i.e., from strictly paired designs such as permanent plots or calculated from a resampling procedure designed to reach a balanced dataset) or if data were collected opportunistically (i.e., resurveys conducted within the same general area, but not necessarily balanced);
- Underlying transformation of the data (referred to as *raw*), indicating whether the estimated range shift was derived directly from the data (i.e., raw with little to no data cleaning) or if estimates were derived from models (e.g., occupancy models).

Studies that did not provide sufficient information to assess these metrics were excluded from the review. For all studies, a second reviewer independently read the article and checked the five points listed above for study validity assessment. Differences identified by the second reviewer were addressed by the original paper reviewer, and any disagreements were discussed with a third co-author. By considering these methodological variables, we were able to capture the variety and diversity of methodological attributes specific to each study, and to account for the role of methodological factors in explaining variation in estimated range shifts (see *Data Synthesis & Presentation* section below) [9].

Additional observations regarding the study design and findings, such as missing data or unreported outcomes, were noted. Corresponding authors were contacted to obtain the missing information, and studies were excluded if authors did not respond or if the absence of the data prevented complete entry into the database.

### Data coding and extraction strategy

Data from each article that passed title/abstract and full text review were extracted and entered into a spreadsheet that was initially shared among co-authors in Google Drive. This table was converted to an excel file (Additional File 3). Range-shift data were extracted for each species described in an included study; therefore, if a study contained results for more than one species, that study had more than one row in the table. In addition, for a given taxon in a given study, we reported range-shift values on distinct dimensions, time periods (e.g., shifts were reported separately between a baseline survey and resurvey, and between the first resurvey and the second), and range-shift parameters on separate rows, if the authors reported these shifts as disaggregated data. We kept time period data disaggregated to avoid re-calculating range shifts produced by the authors, and also to allow future assessments on differences in shifts across time periods (e.g., looking at differences in climate exposure between different study periods). Each entry included an attribute to identify its source (i.e., an article identifier).

**Fig. 2:**
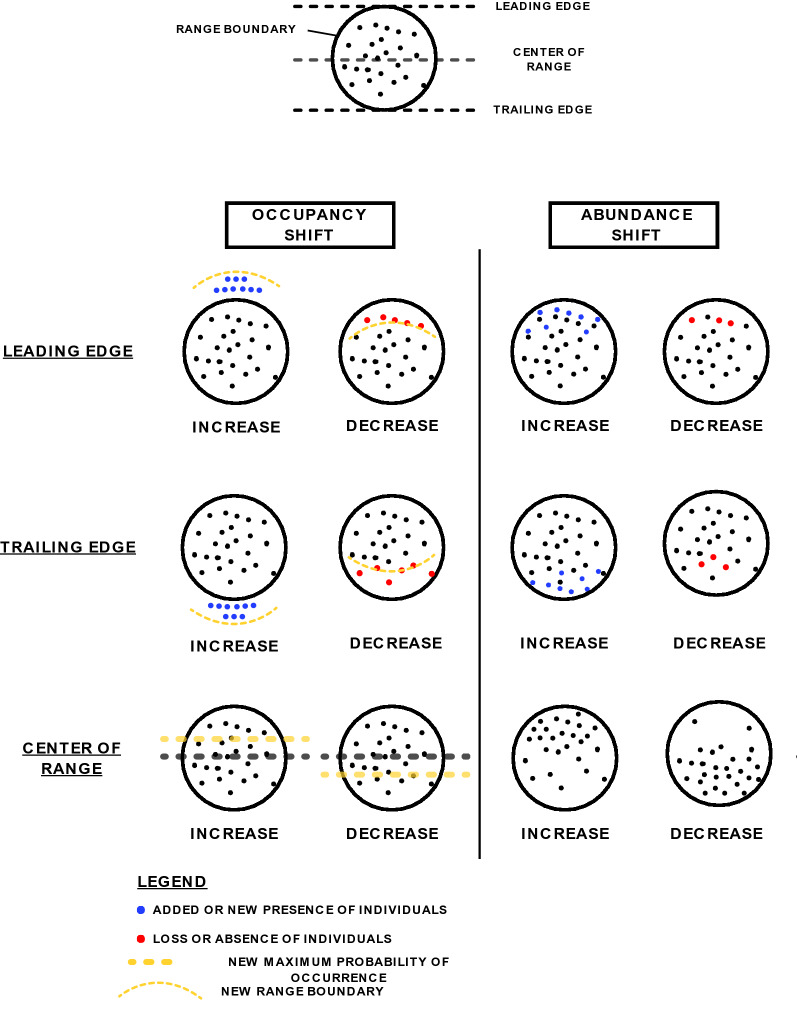
Typology of range shifts used in this review. Leading edge or center of range increases and trailing edge decreases were considered to support our temperature-related range shift hypotheses. Lack of shifts in any parameter; decreases on the leading edge or in the mean/optimum; and increases on the trailing edge were all considered “fail to support”

Broadly, we extracted data from each study into four general categories: (i) basic information about the study itself (i.e., study duration, study location, methodological factors); (ii) basic information on the species included in the study, including scientific names and taxonomic grouping/information (see also 5,6); (iii) information about the observed range shift response (e.g., latitude/longitude/elevation/depth, shift in occupancy or abundance, leading/trailing edge) [8]; and (iv) description of the shift, including both direction (e.g., latitudinal increase, elevational decrease), and, when available, magnitude (expressed as kilometers per decade) (see Additional File 4 for full description of metadata). We considered a study to have observed a range shift if the study’s author(s) found a change in location of the leading or trailing edge (i.e., occupancy shift on the leading or trailing edge), a change in abundance on the leading or trailing edge (i.e., abundance shift on the leading or trailing edge), or a shift in maximum abundance or probability of presence within the range or part of the range (i.e., center-of-range shift) (Fig. 2). We used the range position as defined by the author(s) of the study (e.g., leading edge defined as the 95th percentile of the latitudinal occurrences or the northernmost latitudinal occurrence, center-of-range as the median of the latitudinal occurrences or maximum probability of presence). For all studies, we indicated whether the observation supports or fails to support the hypothesized range-shift expectations. For example, observations of latitudinal decrease or no change in latitudinal distribution would be categorized as “*fails to support*” given the general expectation of poleward shifts.

#### Database review & logical checks

We ensured reproducibility and consistency in data coding and extraction by writing a detailed manual of instructions and guidelines on the process of entering data into our spreadsheet (Additional File 4). This was detailed enough for any of our co-authors to follow. It included complementary information clarifying our eligibility criteria and it answered common questions encountered during the review process. In addition, we trained reviewers to ensure consistency in how data were entered. We implemented a dual-review system so that all articles and associated data entry were reviewed by a second co-author. To resolve any questions or problems, the authors convened and reviewed questionable entries.

After our initial data entry, we performed additional data validation and cleaning steps in R version 4.0.4 [43]. We performed a series of logical checks to confirm that range dimensions, categorical changes, hypothesis support, and sign of numeric shifts aligned for all entries; for example, an observation categorized as “latitudinal increase” should have a positive numeric entry (positive numbers indicating movement towards the poles) and be categorized as “supports”. We ensured that species names used in the database were correct and up to date using the taxonomizr package [44]. We verified species names and pulled the full taxonomic classification for each species from the National Center for Biotechnology Information (NCBI [45]), Global Biodiversity Information Facility (GBIF [46]), Integrated Taxonomic Information System (ITIS [47]), Arctos [48], and iNaturalist databases [49]. For species names that did not match any database, we pulled the full taxonomic classification based on genus. Following taxonomic harmonization, we grouped some classes into higher order groups for analysis (e.g., mollusks, crustaceans, plants).

### Potential effect modifiers/reasons for heterogeneity

To better understand the variation in species responses, we extracted and considered several potential sources of variability (i.e., effect modifiers) from each study [8, 9, 42]. Basic information on the study location (e.g., northern vs. southern hemisphere), ecosystem type (terrestrial, freshwater, and marine), and taxonomic group likely contributed to some variation in observed results, since regional and taxonomic variation in climate-change-driven range shifts is expected [7, 22, 50]. Information on study methodology and data quality (see study validity assessment section above) was also included as potential effect modifiers [8, 9, 42].

### Data synthesis & presentation

First, we calculated basic summary statistics on the database (e.g., total number of range-shift records, total number of species registered, geographic regions covered). We then assessed the direction and magnitude of range shifts. We determined that studies which measured range shifts over fewer than 10 years should be excluded due to the potential for confounding effects from short-term and decadal climatic variability (e.g., temporary range shifts arising from decadal climate phenomena such as ENSO) rather than long-term anthropogenic climate change. This resulted in the exclusion of twenty-four articles that were otherwise eligible for inclusion in the review. For taxon-specific analyses, we excluded taxonomic classes with fewer than 100 observations or for which only one study described the range shift. We grouped most organisms by class, but grouped a few at higher taxonomic levels that might be relevant for managers if it allowed the group to have over 100 observations (e.g., plants, molluscs, and crustaceans).

#### Direction

In this portion of the analysis, the response variable was a binary factor describing the observation as either supporting or failing to support general range shift expectations. For temperature-related analyses, we considered all increases in latitude, elevation, and depth (via distribution or abundance) as “support”, given that they conform to general range shift expectations; all decreases or lack of statistically significant movement were categorized as “failed to support.” In cases where statistical significance was not assessed (e.g., only qualitative shifts were reported), we based our support/fail to support assessment on the overall direction of shift reported by the authors. Because similarly broad expectations about longitudinal range shifts are not available (i.e., there is no a priori expectation of westerly or easterly movement to track isohyet or isotherms), we did not include longitudinal shift data in temperature-related analyses. Instead, we included longitudinal shifts only in precipitation-related analyses when authors expressed a specific isohyet-tracking hypothesis, and we followed the individual author assessment of whether species conformed to the hypothesis: that is, we generally expected species to move longitudinally to follow their precipitation niche, and we relied on the individual author assessment of whether the species conformed to that hypothesis. Therefore, observations which tracked an isohyet were categorized as “support,” and those that did not were categorized as “fails to support.”

To assess the probability that observations support/fail to support general range shift expectations related to temperature, we fitted binomial logistic regression models using the glm function from the stats package in R [43]. We fitted separate models for each explanatory variable of interest (i.e., range dimension, parameter, taxonomic group, and ecosystem type), because some of the variables are related (e.g., the taxonomic group fish could only be in freshwater or marine ecosystem types) and fitting separate models allowed us to more clearly explore these relationships. Each of these variables of interest, as well as the five methodological variables were used as fixed effects (see Additional File 5: Table S2 for full description of the models) [8, 42]. After fitting the logistic regressions, we used the emmeans function in the emmeans package [51] to compute the estimated marginal mean (EMM) probability and 95% confidence interval of support for general range (i.e., the predicted probability of support/fails to support after averaging across the methodological variables weighted proportionally to their occurrence in the original dataset). We tested for significant differences in EMMs (p < 0.05) across variables of interest using a post-hoc Tukey adjustment.

#### Magnitude

We used regression analysis to assess the magnitude of range shifts on the quantitative observations in our database (i.e., observations expressed as unit space per unit time). We did not use random-effects meta-analysis, because it is difficult to rigorously define study sample size in this context (e.g., number of sites in the study, number of years used to estimate range shifts, number of individual surveys, or number of species). In most cases, these data are not reported, and given the various ways that studies measured range shifts, sample sizes in this sense would not be directly comparable. Thus, we have limited confidence in the comparability and consistency of sample size measurements across studies to use this as a weight for observations in a regression model. Instead, we converted all quantitative measures of range shifts to a standardized unit (kilometers per decade) for ease of comparison across dimensions, and with previous meta-analyses (raw and converted shift values are included in Additional File 3). We calculated the average velocity of range shift by dimension (e.g., overall latitudinal, elevational, and depth shifts), as well as for particular subsets of observations (e.g., by range parameter and taxonomic group).

To account for methodological factors, we first fit a multiple linear regression with methodological variables as predictors of km/dec shifts to assess the significance of methodological variables on quantitative estimates of range shifts, and to gain a general understanding of the direction and magnitude of the relationship between methodological factors and the magnitude of range shifts (see Additional File 5: Table S2 for full description of statistical models). Having established that all methodological variables contributed significant variation to quantitative range-shift estimates (Additional File 5: Table S3), we calculated estimated marginal means (EMM; as above) to estimate range-shift coefficients for specific taxonomic groups using the emmeans package in R [8, 9, 42]. We used post-hoc Tukey adjustments to assess the significance of the marginal means over the methodological variables and used proportional weights when accounting for observations within each methodological category.

In addition, we created a suite of linear mixed-effects model (package lmer) to assess the coefficient size and significance of ecological factors (i.e., dimension, parameter, taxonomic group, and ecosystem type) while controlling for methodological factors. The five methodological factors (see *study validity assessment* above) were set as random effects (i.e., random intercepts; we tested for the significance of random slopes but this did not improve model fit). We used the lmerTest package to estimate p-values for the fixed effects, and the MuMIn package to calculate conditional and marginal R2. We selected our final models by comparing AIC scores. We first determined which methodological predictors to include in the initial modeling suite based on significance in a multiple linear regression with km/dec as the dependent variable and all methodological variables as independent variables. All five methodological variables were significant, and were therefore included in the linear mixed effects model as random effects. In our final model, we included dimension, parameter, taxonomic grouping, and ecosystem type as fixed effects (all were significant based on estimated p-values), and included the suite of methodological variables described above as random effects. Finally, we ran a sensitivity analysis to evaluate the role of statistical outliers in our dataset (Additional File 5: Table S4).

## Review findings

### Review results

Our literature search workflow yielded a total of 94,672 candidate articles from *Web of Science*, *Google Scholar*, and *Scopus*. We reviewed 11,581 of these candidate articles before reaching the peak of the accumulation curve during the abstract and title review phase. Another 519 articles were identified through our additional search methods, updating our total articles reviewed to 12,100. Of these, 842 candidate articles passed the initial title/abstract review for in-depth review. Full-text review resulted in a final list of 315 articles fitting criteria for inclusion in the database (Fig. 1; see Additional File 2 for list of all reviewed/included articles and full study validity assessment). These numbers do not include duplicate articles, since Colandr removes duplicates before abstract screening. Of the 315 articles included in the final database, 124 came directly from online database searches, while the remaining 191 articles came from review paper search, snowball methodology, and opportunistic articles. This is likely due to the order of our search procedure, and the prominence of previously published high-profile review papers. We initially reviewed only 2000 abstracts from our database search before beginning the snowball methodology. Included within this original 2000 abstracts were several large, high-profile review papers which cited many relevant primary-source articles. These articles are therefore coded in our database as coming from “snowball methodology”, even though they also turned up later inour extended database search as well. We completed the article searches in: Web of Science on April 16, 2019; Google Scholar on June 14, 2019; and Scopus on December 17, 2019. Opportunistically retrieved articles were accepted through June 2021.

### Distribution of database observations

**Fig. 3:**
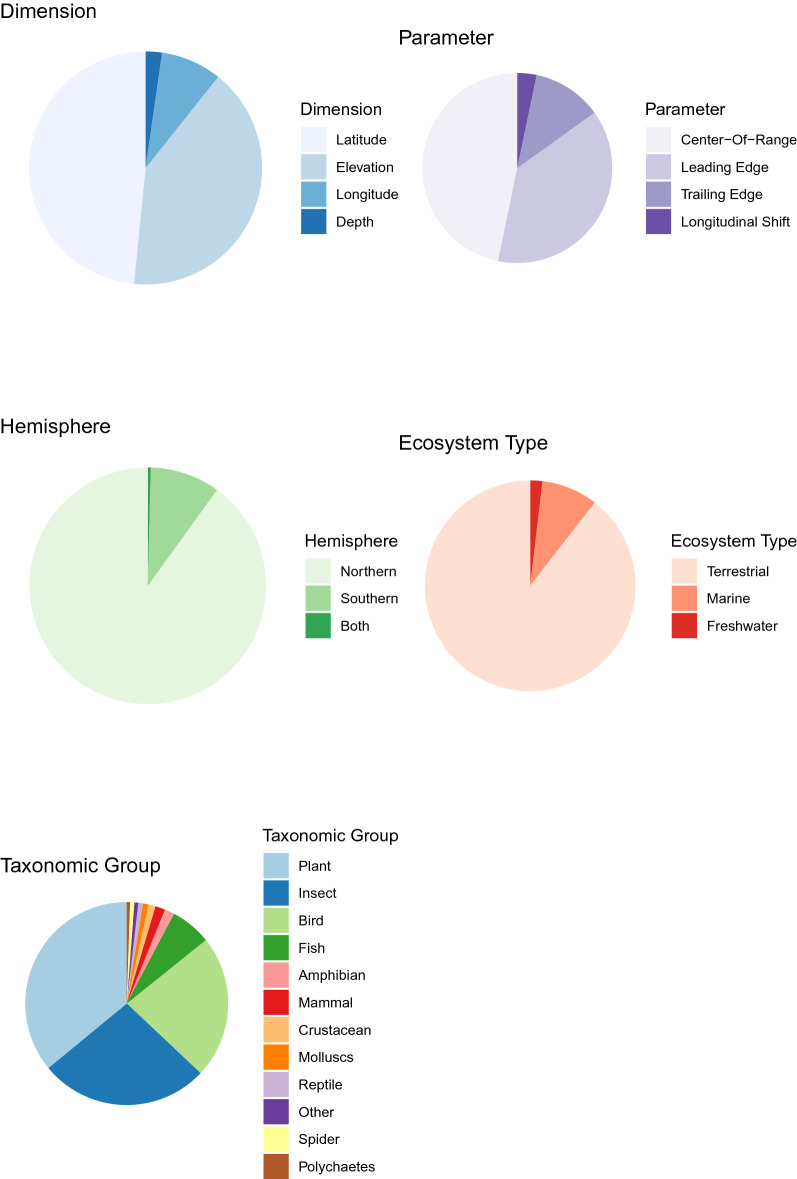

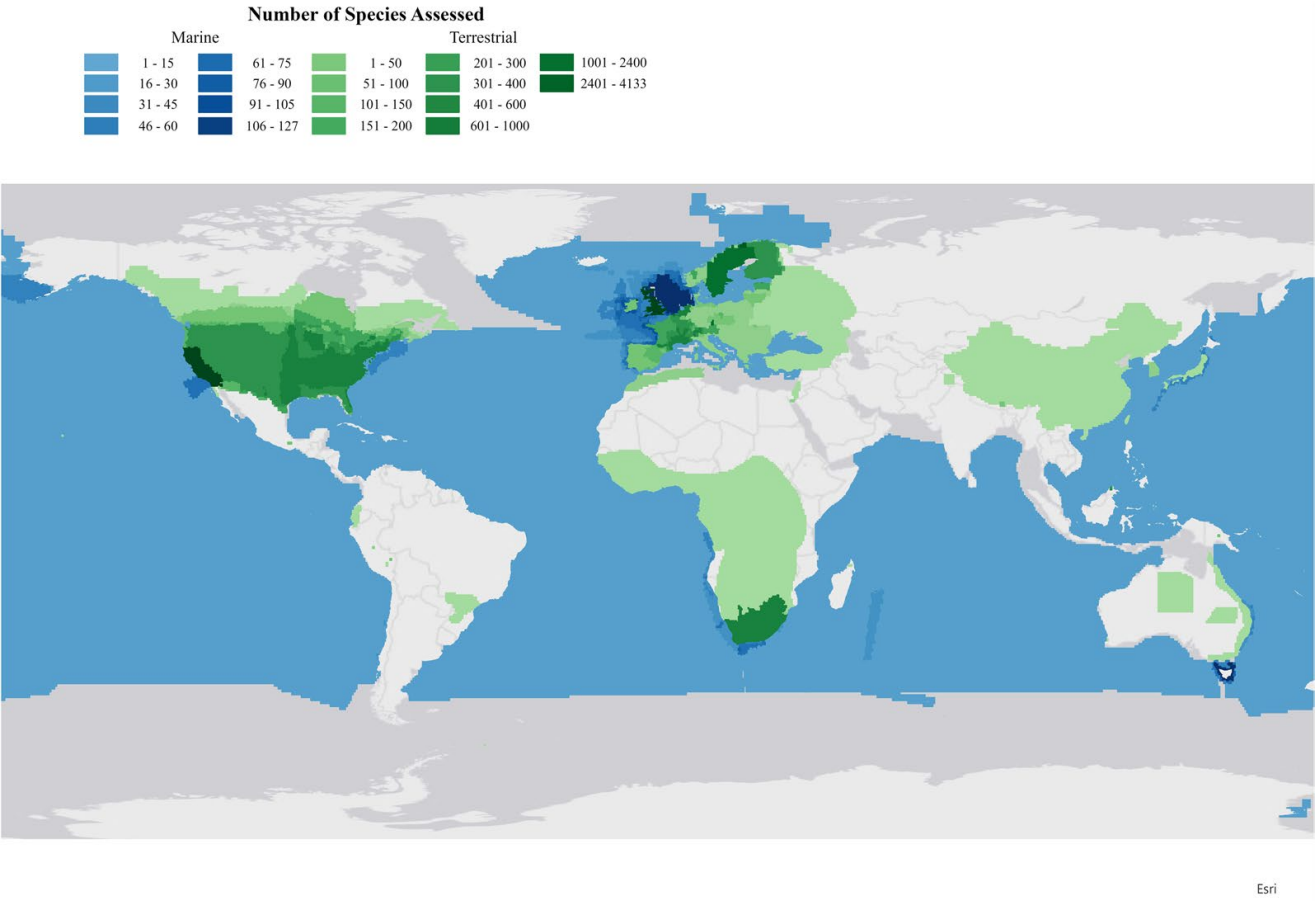
** a** Distribution of observations in the Database. An evaluation of how observations are distributed in the database, including by dimension, parameter, hemisphere, ecosystem type, and taxonomic group. Note: longitudinal shifts were considered only for precipitation-related analysis. **b** Global Distribution of Species in the Database. Counts reflect number of species assessed, rather than individual studies, represented in 1degree cells. Darker colors reflect more species

The 315 articles included in the final database reported 32,632 range-shift estimates across 12,009 species. The number of estimates per study (i.e., the number of rows entered for the paper based on criteria described above) ranged from one to 4426, with a median of 11. A few studies included very large number of estimates (10 articles had over 500). These studies included multiple species and multiple estimates per species (e.g., leading edge and trailing edge, multiple time periods, or multiple study areas). Range-shift estimates were heavily concentrated in the Northern Hemisphere (90.06%), with only 9.5% of estimates occurring in the Southern Hemisphere and the remainder occurring across both hemispheres (Fig. 3a, b, Additional File 5: Table S5). Range-shift estimates were roughly evenly split between latitudinal (48.4%) and elevational range shifts (40.90%), with depth and longitudinal range shifts representing a small proportion of the registered estimates at the species level (2.26% and 8.43%, respectively; Fig. 3a, Additional File 5: Table S5). Center-of-range and leading-edge estimates (46.75% and 38.1%, respectively) were more common than range-shift estimates at the trailing edge (11.83%). The vast majority (89.48%) of estimates were terrestrial, with only 8.6% coming from marine environments and even fewer from freshwater environments (1.88%, Fig. 3a, Additional File 5: Table S5). As a percentage of overall estimates, plants (35.96%) and some groups of animals, including birds (22.81%), insects (27.01%), fish (6.46%), and mammals (1.54%) were well represented (Fig. 3a, Additional File 5: Table S5). These biases are more skewed when compared to the total number of species in each of these groups (e.g., there are many more species of plants and insects than mammals or birds).

### Direction of shift

#### Support for temperature-related expected range shifts

**Fig. 4:**
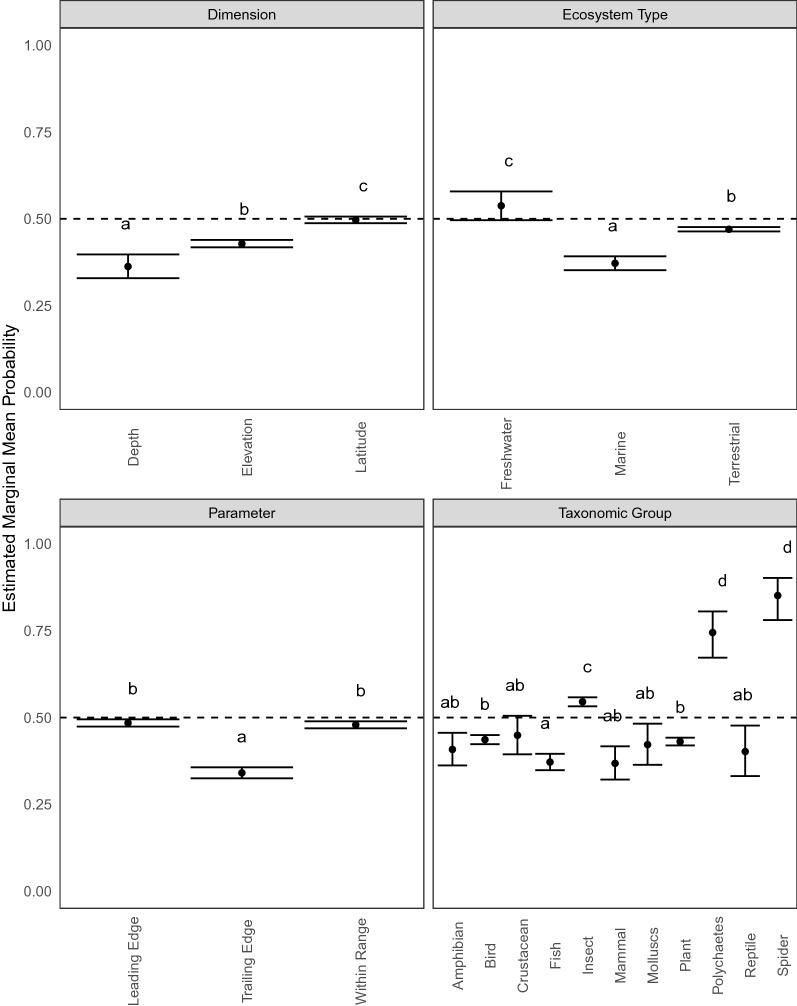
Model Results for temperature hypotheses. Estimated marginal mean probability and 95% confidence intervals of support for shifts poleward, upward, and deeper across dimensions (top left), ecosystem types (top right), parameters (bottom left), and taxonomic groups (bottom right); i.e., the predicted probability of support/fails to support after averaging across the methodological variables weighted proportionally to their occurrence in the original dataset. Letters show significant differences (p = 0.05) across within a given plot using post-hoc Tukey correction

We removed longitudinal shifts for temperature-related analyses, leaving us with 29,881 range shift estimates. Less than half of all the raw observations supported common range-shift expectations (i.e., towards the poles, higher elevations, deeper depths; 46.63%). Estimated marginal means from our logistic regression models showed that there was variation across dimensions, with greater support for shifts to higher latitudes (49.7% of all latitudinal shifts supported expectations; CI 48.7–50.7) and elevations (42.9% of all elevational shifts supported expectations; CI 41.8–43.9) than to deeper depths (36.3% of all depth shifts supported expectations; CI 32.9–39.8; Fig. 4, Additional File 5: Table S6). Support for common range-shift expectations was greater at the leading edge (48.4%; CI 47.4–49.5) and at the center-of-range (47.9%; 46.9–48.9) than at the trailing edge (34.1%; CI 32.5–35.7) (Fig. 4, Additional File 5: Table S6).

Support for common range shift expectations varied across ecosystem types and taxonomic groups. Support for expected shifts was greater than 50% for freshwater systems, less than 50% for terrestrial ecosystems, and lowest in marine ecosystems (Fig. 3, Additional File 5: Table S6). Across taxonomic groups, spiders, polychaetes, and insects showed the greatest level of support for expected shifts (Fig. 4, Additional File 5: Table S6).

#### Precipitation-related expected shifts

Most studies (82.2% of observations) did not assess whether observed range shifts occurred towards geographical locations that would allow the species to maintain their precipitation niche under climate change. Of the remaining observations, failure to support (86.2%; including both observations of no shift or counterintuitive shift) was more common than support (13.8%). All studies that assessed precipitation hypotheses were from terrestrial ecosystems, and nearly all (98%) looked at elevational shifts. Given the small number of observations, we did not run logistic regression models for precipitation.

### Magnitude of shift

**Fig. 5:**
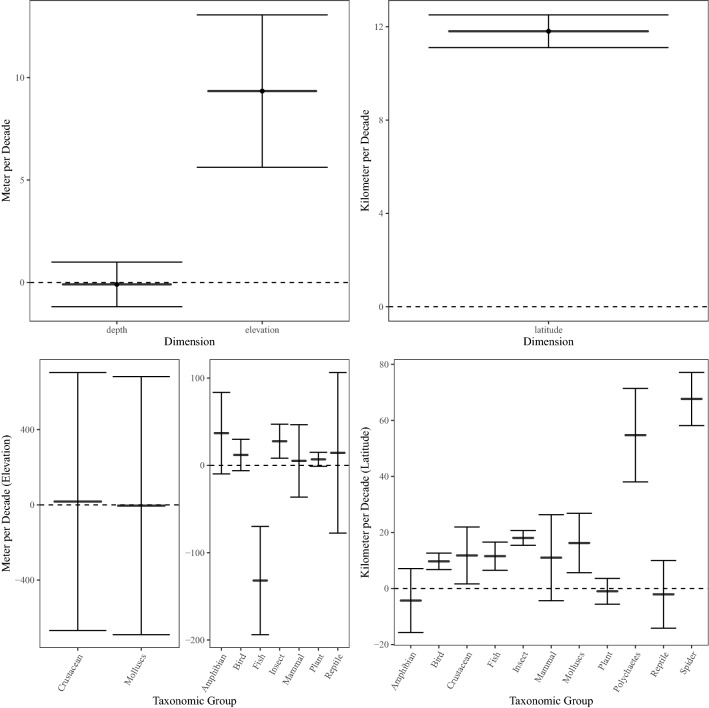
Magnitude of Shift. Estimated shifts by dimension (depth, elevation, and latitude) and by taxonomic group. Estimates by dimension (top row) show average range shifts by dimension, with 95% confidence intervals derived from single-dimension one-sample t-tests (Additional File 5: Table S7). Estimates by taxonomic group (bottom row) display estimated marginal means and associated 95% confidence intervals (Additional File 5: Table S8)

Of the non-longitudinal shift estimates, 25,445 included range shift rates. We found significant rates of shift towards higher latitudes (11.8 km/dec; 95% CI 11.10–12.51; p < 0.05) and higher elevations (9 m/dec; 95% CI 0.0056–0.013, p < 0.05) but a non-significant shift in depth (− 0.09 m/dec; 95% CI − 0.0012–0.001, p-value = 0.8679; note that negative sign indicates shallower movement) (Fig. 5; Additional File 5: Table S7). There was also significant variation by parameter for latitudinal shifts: leading-edge shifts (19.7 km/dec) exceeded center-of-range (4.2 km/dec) or trailing-edge shifts (0.5 km/dec); these parameters are all significantly different from each other when assessed in a multiple linear regression (p < 0.05) (Additional File 5: Table S7). Elevational shifts varied by parameters but these differences were not significant. Finally, we did not assess differences in parameter for depth due to limited observations in this dataset.

We found that all the methodological variables listed in the study validity assessment section had a significant effect on quantitative estimates of range shift (Additional File 5: Table S3). A linear mixed-effects model combining all dimensions with methodological factors as random effects confirmed support for latitudinal poleward shifts (18.4 km/dec, p < 0.05) and upslope shifts (6 m/dec,p = 0.03), even after accounting for methodological shifts. This model found a small (non-significant) average movement towards shallower marine depth of (− 0.19 m/dec, p-value = 0.32)). (Additional File 5: Table S9).

The magnitude of range shift also varied by taxonomic group (Fig. 5, Additional File 5: Table S8). Latitudinal shifts in birds, insects, fish, crustaceans, polychaetes, and spiders were statistically significant (i.e., the 95% confidence interval did not include 0), whereas shifts in reptiles, plants, mammals, and amphibians were not (Fig. 5, Additional File 5: Table S8). For elevational shifts, only two taxonomic groups demonstrated statistically significant shifts: insects shifted significantly upslope, whereas fish shifted significantly downslope (Fig. 5, Additional File 5: Table S8). All other taxonomic groups demonstrated shifts, but these were non-significant (Additional File 5: Table S8).

Finally, we assessed the relative contribution of methodological and ecological variables in explaining overall variation in magnitude of range shifts through a linear mixed effects model in which methodological variables were set as random intercept terms. We followed the methodology of Lenoir et al. 2020, whereby we compared conditional R2 (total variance explained) to marginal R2 (variance explained by fixed effects) to compare the relative ability of methodological vs ecological factors to explain variation in the database. Our final model, which included dimension, parameter, taxonomic group, and ecosystem type as fixed effects, and all methodological variables as random effects had a total variance explained (conditional R2) of 14.3%, while variance explained by ecological (fixed) effects (marginal R2) was 11.9%. Variance explained by methodological variables (conditional R2-marginal R2) was 2.4% (Additional File 5: Table S9). We found that dimension, parameter, taxonomic group and ecosystem type were all statistically significant even after accounting for the variation due to methodological factors.

## Review findings summary

Support for expected temperature-driven range shifts was mixed, with less than half of all observations shifting as expected. This suggests that general expectations of shifts poleward, to higher elevations, and to greater depths cannot be assumed, and that other processes are contributing to global redistribution of biodiversity. Although warming has been observed at the macro-scale, the complex mosaic of local climate velocities may mean that more localized range-shift directions are not necessarily oriented poleward, upward, or bottomward (e.g., [53]). Indeed, local or regional changes in temperature and precipitation patterns, such as isotherms shifting towards the equator or isohyets shifting towards lower elevations, may lead to seemingly counter-intuitive range shifts equatorward for fish (e.g., [53]) and downslope for plants (e.g., [2]), respectively. Changing temperature and precipitation patterns can interact to affect overall water availability, and so considering the multi-dimensional impacts of climate change can lead to different expectations. For example, amphibian species are highly sensitive to changes in temperature and water availability; expected snowpack reductions in the eastern US may reduce species occupancy, while milder winters may improve overwinter survival [54].

In addition, there are numerous important non-climate drivers and disturbances which affect species’ spatial distribution, including land use change, habitat loss and fragmentation, and disturbance regimes such as wildfire and extreme drought [55–58]. Lack of shifts could indicate that species are not keeping pace with the rate of climate change. This could be driven by lags in dispersal or establishment at the leading edge or lags in extinction at the trailing edge [59]. Alternatively, it may indicate that some species are able to adapt in place, such as by changing behavior or phenology [60]. However, even species that are able to adapt phenotypically may do so at a pace that is too slow to allow persistence in the long term [61].

Other range-shift expectations have also been proposed in the scientific literature, including that species ranges may respond more strongly at the leading edge of the range due to higher exposure to temperature changes, especially nearer the pole in the Northern Hemisphere [8, 19, 20, 62]. Abiotic factors might be more important drivers of range limits at leading edges than trailing edges, so species may be able to tolerate warmer conditions than they currently experience [63]. Biotic factors can also interact with abiotic factors to mediate range limits [64]. This can lead to disequilibrium between climatic tolerances and where species are currently found [63]. Methodological factors, however, may contribute to the expectation of faster leading edge shifts, including the fact that colonization at leading edges is easier to detect than extirpations at trailing edges [28]. Indeed, we found greater support for hypothesized range-shift expectations along leading edges compared to trailing edges. We found varying levels of support for the hypotheses among different taxonomic groups. For example, insects showed a high proportion of supporting observations compared to many other taxonomic groups. As small, ectothermic animals that often have short life cycles, insects are especially sensitive to changes in temperature [65, 66].

For magnitude of shifts, we found evidence for an average range shift in latitude and elevation in the direction anticipated (i.e., poleward latitudinal shifts, upslope elevational shifts), but failed to find evidence for an average shift towards greater marine depths. Our dataset demonstrates tremendous variation in observed species’ range shifts, including variation across parameter and taxonomic group. Interestingly, leading edge shifts were faster than trailing edge shifts for latitude, but not for elevation. Similarly, Rumpf et al. [62] found that leading and trailing edges did not differ in rates of shift for elevation. Trailing edge shifts might keep pace with leading edge shifts for elevation rather than for latitude due to increased human pressure on lower elevation sections of mountains, or differences between low and high elevations in the magnitude of climate change or the importance of biotic interactions. Moreover, area for expansion on the leading edge in high elevation areas is limited [62, 67]. In addition, we found that methodological factors accounted for a substantial amount of additional variation, revealing that the techniques used to study range shifts can have a substantial impact on ecological signal. Our review results were comparable to previous range shift meta-analyses (Additional File 5: Table S10). We found similar latitudinal shifts as Chen et al. [7] and Lenoir et al. [9] (in terrestrial environments); our estimates for marine shifts were larger than terrestrial environments (as expected due to fewer barriers to movement and greater thermal connectivity in water; [9]) but smaller than recent analyses in [9]. Our estimates of elevational shifts were similar to [9].

Surprisingly, we found that even though the direction of shift did not support general expectations in many cases, we still found significant rates of shift in expected directions when we looked at the subset of articles reporting range magnitudes. For example, we found less support for direction of range shifts in marine ecosystems compared to terrestrial and freshwater ecosystems, even though, similar to Lenoir et al. [9], we found that rates of marine shifts were faster than terrestrial shifts. This might be explained by observed localized marine climate isotherm shifts, which display greater spatial variability and do not always closely match the coarse hypothesis of poleward/equatorial shifts [53]. It is also possible that even though marine species are shifting more quickly on average, there is high variability in the number of species that are shifting in the expected direction. This seems likely given that we found many marine taxa (e.g., polychaetes, molluscs, fish, and crustaceans) with significant rates of shift towards the poles (Fig. 5).

Differences in methodology between our direction of shift and magnitude of shift analyses may help account for the discrepancy. First, in our direction of shift analysis, we considered studies that qualitatively presented range shifts, even if no quantitative value was provided, whereas our magnitude of shift analysis only included quantitative shifts. Second, our direction of shift analysis included shifts in both occupancy and abundance (i.e., abundance increases or decreases) on leading and trailing edges, and abundance shifts tended to show lower support for expected shifts. Third, in our direction of shift analysis, we considered both no shifts and shifts towards the equator and downslope as “failed to support”. Therefore, no shift observations might have a stronger effect on the direction of shift analysis than the magnitude of shift analysis, because if you have some species moving quickly in one direction (e.g., towards the poles), it could give you an average rate of shift in the direction you expect, even if less than half of all species are shifting. Finally, in some cases, studies reported small quantitative shift values that were not found to be significant due to variability in the data. In these cases, we considered it as “no shift” for the direction analysis but did include the shift value in the magnitude of shift analysis. In some cases, non-significant shifts could reflect data limitations rather than lack of shifts. This could mean that quantitative shifts might be a better earlier indicator of response and that shifts might accelerate in the future.

Even though on average, rates of shift show significant movement to higher elevations and latitudes for many taxa, less than half of species are shifting in these directions. In order for managers to effectively plan for species shifts, we need to better account for and predict which species will shift and by how much.

### Influence of methodological factors

Similar to other findings [9, 42], we found that methodological variables play an important role in explaining overall variability in range shifts. As in Brown et al.[42], we found that studies using irregular time sampling estimated greater rates of change than regular sampling; this is likely due to infrequent sampling conflating short-term variability with long term trends. We also found that estimated range shifts were lower in studies using abundance data rather than occurrence data—this may be because changes in occurrence are more readily observable, whereas shifts in abundance require greater monitoring, reflecting the greater sensitivity of abundance data in reflecting early stages of range shifts [9, 68]. Additionally, studies examining more than one species reported smaller range shifts than single-study species; this may be due to publication bias towards single-species studies where range shifts are expected or easily observed [41]. While Brown et al. [42] and Lenoir et al. [9] found that variance explained by methodological factors exceeded variance explained by ecological factors, we found that more variability was explained by ecologically relevant factors like dimension, parameter, taxonomic group and ecosystem type. This discrepancy may reflecte differences in methodologies: Lenoir et al. [9], for example, considered family and genus to be random effects rather than fixed ecological effects in their analyses.

### Review limitations

We only searched for relevant articles in English in our review, and we included only 2 non-English articles found during our snowball search (one in Spanish and one in French). This may have introduced geographical bias in our search results, but it circumvented the difficulties and delays associated with translation of materials. In the future, our methodology could be conducted using a greater breadth of studies in other languages and evaluated by fluent speakers. In addition, we limited our review to observed, long-term range shifts, but excluded experimental studies or those documenting shifts in response to short-term changes in temperature or precipitation. We chose to exclude these studies to focus on evidence for long-term range shifts observed thus far, but such studies could provide useful information on mechanisms driving shifts. Finally, we limited our review to articles providing species-level range shift information, but excluded many articles documenting assemblage-level changes.

Limitations in the underlying articles may have affected our review conclusions. First, the number of observations for different range dimensions, parameters, and taxonomic groups was skewed. We had fewer depth or longitude observations than latitude or elevation, and trailing-edge observations were less common than leading-edge or within-range shifts. Plants, insects, and birds were studied far more often than other taxonomic groups. In addition, very few studies assessed range shifts in comparison to historical variability (i.e., used continuous data or used multiple years of data to establish baseline and current range-shift estimates). Range edges are dynamic [52], so studies that do not account for year-to-year variability may overestimate shifts.

In addition, it is extremely difficult to quantify measures of uncertainty for range shift observations as reported in most of the literature we reviewed. Direct measures of uncertainty are rarely provided in range shift studies, which tend to report only absolute changes in range boundaries or mean distributions and do not estimate how these estimates are affected by sampling distributions or observational error. Lack of standard error estimates in the underlying literature prevented us from conducting a formal assessment of publication bias. Therefore, the overall estimates of effect size presented in this review do not explicitly account for measures of uncertainty or confidence intervals at the scale of individual articles. Including measures of uncertainty in range shift studies would allow us to better assess our confidence in range shift estimates: in particular, it would be valuable for studies to assess the degree of historical variability in range shift boundaries in order to contextualize the significance of contemporary range shifts with respect to baseline variability.

An additional limitation is that there may be some degree of correlation between observations of the same species, or phylogenetically similar groupings, in our review. While we expect exposure to be a primary determinant of species observed range shifts, we may also expect that species which are more phylogenetically related should demonstrate more similar responses, resulting in a lack of independence of these observations. While we cannot account for species-level dependence directly in our models due to model convergence issues, we do include taxonomic grouping as an explanatory variable when calculating overall effect size, which accounts for some of this correlation.

Finally, our inclusion of longitudinal range shift observations was only useful to evaluate precipitation-related hypotheses if the study explicitly reported the direction of precipitation changes. This meant that we had to record many longitudinal shifts (99%) as indeterminant regarding their support for this hypothesis. However, if no shifts occurred but changes in temperature or precipitation were reported, we were able to record shifts as “fail to support”; “fail to support” observations are therefore likely over-reported in our review.

## Review conclusions

### Implications for research

In this review, we assessed whether established range-shift hypotheses were supported by available evidence. However, we did not explicitly link observed range shifts and climate change. Other range-shift analyses have done this by associating shifts with trends in temperature or other ecologically relevant climate variables; others by calculating alignment or mismatch with climatic isotherms (i.e., climate velocity). Although these metrics are useful indicators, they do not assess whether observed changes are tracking local climate velocities or whether they are significant compared to historical climate variability, and therefore whether the species is experiencing changes outside of what is “normal” for its range. To address this limitation, future range-shift studies should consider comparing changes to baseline data on historical range variability. Future studies may consider using a signal: noise ratio analysis to assess whether exposure to climate change is historically significant, and if so, the time of signal emergence [69, 70]. Additionally, species may be responding to changes in multiple climate variables that may not be shifting the same way [1]. Few studies in our review assessed whether species shifts followed precipitation changes. Considering a variety of temperature and precipitation variables could improve our ability to predict shifts.

In addition to considering species exposure to climate change, assessing species sensitivity and adaptive capacity may help to explain varying responses [60, 71]. Thus far, studies have not found strong support for specific traits as predictors of species range shifts [72–74]. However, accounting for exposure, sensitivity, adaptative capacity, and non-climate drivers such as land-use change together in a vulnerability framework approach may prove more fruitful [75]. This database could form the basis for such an analysis. Many articles included in our review discussed traits or other drivers that might explain why species shifted as they did. We captured these explanations qualitatively in our database. Conducting a full text analysis of this section could provide a useful starting point for identifying key traits or drivers. From an initial screening, some of the non-climate drivers included land use change or habitat loss, changes in habitat quality, changes in anthropogenic pressure such as exploitation or disturbance, and biotic interactions such as competition or predation. Traits included behavioral plasticity, thermal tolerance, dispersal ability, habitat specialization, and life span. Moreover, our data could be used for more in-depth analyses of differences among population subgroups (e.g., male vs. female, age class) that may further help to explain observed variability. Finally, future research efforts focusing on better representing currently underrepresented regions (particularly the southern hemisphere) and taxonomic groups would provide information necessary for understanding global variability. Overall, the dataset produced for this analysis can be used for future research to explore additional hypotheses to better understand species range shifts.

### Implications for management/policy

Species range shifts have the potential to restructure ecosystems, with implications for biodiversity conservation, ecosystem functioning, economic development, and human health and well-being [5]. We have shown that species shifts are highly variable, with many species not shifting as expected. Indeed, our direction of shift analysis, which accounted for changes in both occupancy and abundance, found less support for common range shift hypotheses than when we only considered magnitude of shifts. In some cases, changes in species abundance can be more impactful for ecosystem function and management than changes in occupancy. For example, if only a few individuals have shifted outside their historical range on the leading edge, it may not trigger new management plans, but if the abundance greatly increases, new management plans may be warranted. Additional modeling and monitoring to anticipate and detect species shifts is needed to assess when these species may need to be integrated into management plans [76]. Monitoring the impacts of range-shifting species would also be beneficial, as shifting species can have unanticipated impacts on recipient communities [77]. For example, insects seem to be shifting especially quickly. In general, this could be a positive sign of adaptation, but can be a concern for certain pest or disease carrying species [78, 79]. Because latitudinal shifts are faster on the leading edge than the trailing edge, monitoring may be especially important on the leading edge.

Management planning for climate may require more nuanced examination of evidence. For example, most amphibians, plants, and reptiles are not shifting as expected, and overall rates of shift were not different than zero. For plants, competitive release, or changes in habitat or precipitation/water balance could drive unexpected shifts, at least for elevation [1, 21]. However, non-shifting species may warrant additional management attention. In particular, amphibians are declining globally [80, 81], and so lack of shifts to follow changing climate drivers may be especially concerning.

As species move outside of their current ranges, current management practices may no longer be effective. For example, existing fisheries management is based on assumptions of stationarity—that population ranges are generally stable over time [82, 83]. As species move across sub-national and national borders, transboundary conflicts are possible [82]. Flexible, pro-active, and transboundary management policies may help address this challenge [76].

## Supplementary Information

**Additional file 1: **Search terms and articles used to test the sensitivity of our literature search terms.

**Additional file 2: **List of all articles reviewed and included/excluded in this analysis. Reasons for exclusion are provided where relevant.

**Additional file 3:** Database of species range shifts compiled for this review from articles meeting all inclusion criteria. The database, including study area shapefiles, will also be available for download from the USGS at https://doi.org/10.5066/P99VP2TW. 

**Additional file 4: **Metadata for additional file 3- manual with instructions for data extraction.

**Additional file 5: **Supplemental information on data analysis and results.** Table S1.** Sensitivity Analysis: Search protocol stopping criteria. **Table S2.** Overview of all models used to assess the direction and magnitude of shifts compared to general range shift expectations. **Table S3.** Significance of methodological variables in predicting magnitude of range shift. **Table S4.** Identifying & Evaluating Statistical Outliers. **Table S5.** Distribution of observations in the dataset. **Table S6.** Qualitative support vs fails to support. **Table S7.** Km/decade shifts by dimension (latitude, elevation, and depth) and parameter (leading edge, trailing edge, center-of-range). **Table S8.** Km/Dec Shifts by Taxonomic Grouping. **Table S9.** Relative Contribution of Ecological and Methodological Variables in Predicting Magnitude of Range shift. **Table S10.** Comparison of km/dec shift with previous meta-analyses.

**Additional file 6: **ROSES systematic review checklist.

## Data Availability

The datasets generated through our search and analyzed as part of this systematic review will be made publicly available Science Base (25).
