# Supplementary material for: Climate change and the global redistribution of biodiversity: substantial variation in empirical support for expected range shifts
Source: Environ Evid. 2023 Apr 11;12:7. doi: 10.1186/s13750-023-00296-0 (PMC11378804; doi:10.1186/s13750-023-00296-0)
Supplement: Supplementary file 1 — Additional file 1: Search terms and articles used to test the sensitivity of our literature search terms. [file 13750_2023_296_MOESM1_ESM.docx]

**Additional File 1: Search terms and articles used to test the sensitivity of our literature search terms.**

**Search Terms**

- Web of Science: TS=((“climate” OR “global warming” OR "temperature" OR "precipitation") AND (“range” OR “distribution” OR “habitat extent” OR “occupancy”) AND (“species”))
- Google Scholar: (“climate” OR “global warming” OR "temperature" OR "precipitation") AND (“range” OR “distribution” OR “habitat extent” OR “occupancy”) AND (“species”))
- Scopus: ALL(("climate" OR "global warming" OR "temperature" OR "precipitation") AND ("range" OR "distribution" OR "habitat extent" OR "occupancy") AND ("species"))

**Articles used to test the sensitivity of our literature search terms. All articles were returned in the Web of Science, Google Scholar, and/or Scopus search results.**

1. Archaux F. Breeding upwards when climate is becoming warmer: No bird response in the French Alps. Ibis (Lond 1859). 2004;146:138–44.

2. Bates AE, Pecl GT, Frusher S, Hobday AJ, Wernberg T, Smale DA, et al. Defining and observing stages of climate-mediated range shifts in marine systems. Glob Environ Chang [Internet]. Elsevier Ltd; 2014;26:27–38. Available from: <http://dx.doi.org/10.1016/j.gloenvcha.2014.03.009>

3. Beckage B, Osborne B, Gavin DG, Pucko C, Siccama T, Perkins T. A rapid upward shift of a forest ecotone during 40 years of warming in the Green Mountains of Vermont. Proc Natl Acad Sci U S A. 2008;105:4197–202.

4. Bergamini A, Ungricht S, Hofmann H. An elevational shift of cryophilous bryophytes in the last century - An effect of climate warming? Divers Distrib. 2009;15:871–9.

5. Brommer JE. The range margins of northern birds shift polewards. Ann Zool Fennici. 2004;41:391–7.

6. Buckley LB, Kingsolver JG. Functional and Phylogenetic Approaches to Forecasting Species’ Responses to Climate Change. Annu Rev Ecol Evol Syst. 2012;43:205–26.

7. Chen IC, Hill JK, Ohlemüller R, Roy DB, Thomas CD. Rapid range shifts of species associated with high levels of climate warming. Science (80- ). 2011;333:1024–6.

8. Chen IC, Hill JK, Shiu HJ, Holloway JD, Benedick S, Chey VK, et al. Asymmetric boundary shifts of tropical montane Lepidoptera over four decades of climate warming. Glob Ecol Biogeogr. 2011;20:34–45.

9. Chen I, Hill JK, Ohlemüller R, Roy DB, Thomas CD. Rapid range shifts of species of climate warming. Science (80- ). 2011;333:1024–6.

10. Estrada A, Morales-Castilla I, Caplat P, Early R. Usefulness of Species Traits in Predicting Range Shifts. Trends Ecol Evol [Internet]. Elsevier Ltd; 2016;31:190–203. Available from: <http://dx.doi.org/10.1016/j.tree.2015.12.014>

11. Foden WB, Butchart SHM, Stuart SN, Vié JC, Akçakaya HR, Angulo A, et al. Identifying the World’s Most Climate Change Vulnerable Species: A Systematic Trait-Based Assessment of all Birds, Amphibians and Corals. PLoS One. 2013;8.

12. Franco AMA, Hill JK, Kitschke C, Collingham YC, Roy DB, Fox R, et al. Impacts of climate warming and habitat loss on extinctions at species’ low-latitude range boundaries. Glob Chang Biol. 2006;12:1545–53.

13. Hickling R, Roy DB, Hill JK, Fox R, Thomas CD. The distributions of a wide range of taxonomic groups are expanding polewards. Glob Chang Biol. 2006;12:450–5.

14. Hitch AT, Leberg PL. Breeding distributions of North American bird species moving north as a result of climate change. Conserv Biol. 2007;21:534–9.

15. Holzinger B, Hülber K, Camenisch M, Grabherr G. Changes in plant species richness over the last century in the eastern Swiss Alps: Elevational gradient, bedrock effects and migration rates. Plant Ecol. 2008;195:179–96.

16. Kelly AE, Goulden ML. Rapid shifts in plant distribution with recent climate change. Proc Natl Acad Sci U S A. 2008;105:11823–6.

17. Konvicka M, Maradova M, Benes J, Fric Z, Kepka P. Uphill shifts in distribution of butterflies in the Czech Republic: Effects of changing climate detected on a regional scale. Glob Ecol Biogeogr. 2003;12:403–10.

18. Le Roux PC, McGeoch MA. Rapid range expansion and community reorganization in response to warming. Glob Chang Biol. 2008;14:2950–62.

19. Lenoir J, Gégout JC, Marquet PA, De Ruffray P, Brisse H. A significant upward shift in plant species optimum elevation during the 20th century. Science (80- ). 2008;320:1768–71.

20. Lenoir J, Svenning JC. Climate-related range shifts - a global multidimensional synthesis and new research directions. Ecography (Cop). 2015;38:15–28.

21. Lima FP, Ribeiro PA, Queiroz N, Hawkins SJ, Santos AM. Do distributional shifts of northern and southern species of algae match the warming pattern? Glob Chang Biol. 2007;13:2592–604.

22. MacLean SA, Beissinger SR. Species’ traits as predictors of range shifts under contemporary climate change: A review and meta-analysis. Glob Chang Biol. 2017;23:4094–105.

23. Maggini R, Lehmann A, Kéry M, Schmid H, Beniston M, Jenni L, et al. Are Swiss birds tracking climate change?. Detecting elevational shifts using response curve shapes. Ecol Modell. Elsevier B.V.; 2011;222:21–32.

24. Moritz C, Patton JL, Conroy CJ, Parra JL, White GC, Beissinger SR. Impact of a century of climate change on small-mammal communities in Yosemite National Park, USA. Science (80- ). 2008;322:261–4.

25. Pacifici M, Visconti P, Butchart SHM, Watson JEM, Cassola FM, Rondinini C. Species’ traits influenced their response to recent climate change. Nat Clim Chang. 2017;7:205–8.

26. Parmesan C, Yohe G. A globally coherent fingerprint of climate change impacts across natural systems. Nature [Internet]. 2003;421:37–42. Available from: <http://www.nature.com/doifinder/10.1038/nature01286>

27. Parolo G, Rossi G. Upward migration of vascular plants following a climate warming trend in the Alps. Basic Appl Ecol. 2008;9:100–7.

28. Popy S, Bordignon L, Prodon R. A weak upward elevational shift in the distributions of breeding birds in the Italian Alps. J Biogeogr. 2010;37:57–67.

29. Pöyry J, Luoto M, Heikkinen RK, Kuussaari M, Saarinen K. Species traits explain recent range shifts of Finnish butterflies. Glob Chang Biol. 2009;15:732–43.

30. Raxworthy CJ, Pearson RG, Rabibisoa N, Rakotondrazafy AM, Ramanamanjato JB, Raselimanana AP, et al. Extinction vulnerability of tropical montane endemism from warming and upslope displacement: A preliminary appraisal for the highest massif in Madagascar. Glob Chang Biol. 2008;14:1703–20.

31. Rivadeneira MM, Fernández M. Shifts in southern endpoints of distribution in rocky intertidal species along the south-eastern Pacific coast. J Biogeogr. 2005;32:203–9.

32. Townsend A. SUBTLE RECENT DISTRIBUTIONAL SHIFTS IN GREAT PLAINS BIRD SPECIES Author : Peterson , A . Townsend Published By : Southwestern Association of Naturalists. 2020;48:289–92.

33. Urban MC, Bocedi G, Hendry AP, Mihoub JB, Pe’er G, Singer A, et al. Improving the forecast for biodiversity under climate change. Science (80- ). 2016;353.

34. Wilson RJ, Gutiérrez D, Gutiérrez J, Martínez D, Agudo R, Monserrat VJ. Changes to the elevational limits and extent of species ranges associated with climate change. Ecol Lett. 2005;8:1138–46.

35. Zuckerberg B, Woods AM, Porter WF. Poleward shifts in breeding bird distributions in New York State. Glob Chang Biol. 2009;15:1866–83.
