# Supplementary material for: Climate change and the global redistribution of biodiversity: substantial variation in empirical support for expected range shifts
Source: Environ Evid. 2023 Apr 11;12:7. doi: 10.1186/s13750-023-00296-0 (PMC11378804; doi:10.1186/s13750-023-00296-0)
Supplement: Supplementary file 4 — Additional file 4: Metadata for additional file 3- manual with instructions for data extraction. [file 13750_2023_296_MOESM4_ESM.docx]

**Climate change and the global redistribution of biodiversity: are expected range shifts supported by the evidence?**

**Manual for Systematic Literature Review**

**Paper ID Tab**

- *Paper ID* (Column A)*:* Numerical ID assigned to each research article entered in the matrix that has passed Title/Abstract Review
- *Origin* (Column B)*:* Where the article originated from; Could be one of the three databases we searched (Web of Science, Google Scholar, Scopus), from a review paper or meta-analysis that came up in our database search, from our snowball search method, from a similar study by our colleague Jonathan Lenoir (Lenoir et al, 2020) opportunistically found (papers sent to us by colleagues or found coincidentally through other means), or from our new publication alerts from the databases since our original search.
- *Author* (Column C)*:* Author(s) of the article (abbreviated to “first author” et al when 3 or more authors present)
- *Title* (Column D)*:* Full title of the article
- *Year* (Column E)*:* The year of publication
- *Journal* (Column F)*:* Publication where the article is published
- *DOI ISSN* (Column G)*:* Digital object identifier (DOI) code for the article

**Study Species Data Tab (note – column names for R scripts are provided in second row)**

- *Paper ID* (Column A)*:* number of the paper as entered on the tab “Paper Selection”
- *Study Periods* (Column B)*:* Indicates specific study period(s) for each study (e.g. P1, P2 …). Although many studies have only one period (data were collected from time period 1 and then time period 2, a study may have multiple study periods if time intervals were not the same for each species included in the study. In this case, enter each distinct time period as a separate line.
- *Total Years Studied* (Column C): (End Year-Start Year)+1.
  - If there is a date range in the start and/or end period, use the mid-point of each period to calculate total years studied.
  - If the author(s) provide median years for the start and end period, then use that instead of calculating as above.
  - Note: If start date is unclear (e.g. “pre-1970s”) and you cannot calculate total years studied, you should not calculate km/dec shift. Shifts in these cases can be entered qualitatively.
- *Number of taxa* (Column D): Indicate the number of species or distinct population groups assessed in the study. Coded as “ntax” in SI figures & tables.
  - Will be used in analysis to distinguish single-species studies from multi-species studies, which will be used as an indicator of study quality.
- *Regularity of Sampling* (Column E): If authors compared ranges at multiple time intervals, indicate how regularly they sampled. If range was only sampled twice, put n/a. Coded as “sample” in SI figures & table.
  - *Regular* - authors sampled ranges continuously throughout study period at specified time intervals, such as every year or more
  - *Irregular* - authors sampled ranges periodically throughout study period at irregular time intervals
- *Number of Sampling Periods* (Column F): Indicate the number of sampling events during the study. If number is unclear, just put n/a and we will estimate in R
  - This will be used as a metric of study quality in our analysis
  - Enter “n/a” if there are no clear sampling periods (ie, if the study uses historical data or opportunistic data and discreet samples are not provided)
- *Grain* (Column G): If quantitative data on range shifts was provided, indicate the spatial resolution of the data used to generate range shift estimates
  - *Fine*: Data based on GPS coordinates with spatial resolution of less than or equal to 10 km
  - *Coarse:* Data based on range maps or atlas grids with spatial resolution greater than 100 km
  - *Medium:* Data resolution used to calculate range shifts is between fine and coarse
  - **Note: this is not a measure of the result (i.e., species shifted 100km), but instead is a measure of how *specific* the data is: how precisely did the authors measure the range shift.**
- *Resurveyed or opportunistic* (Column H)*:* Similarity of sample sites in the historical and resurveyed periods. Coded as “resurvey” in SI tables & figures.
  - *Opportunistic:* Resurvey conducted in same general area, but not in same plots or transects
  - *Resurveyed:* Range shifts calculated from paired designs such as permanent plots or data cleaning or resampling procedure carried out to calculate range shifts on a balanced dataset (e.g. resurvey conducted in the same general area but not the same plots and authors made sure number of sampling units in both time periods was identical)
- *Raw or modeled* (Column I)*:* Indicate the level of extrapolation. Coded as “raw” in SI tables & figures.
  - *Raw:* little to no data cleaning, range shifts obtained from pure observations or descriptive statistics
  - *Modeled:* Range shifts obtained from model outputs (i.e. models used to project range beyond survey points, such as species distribution model, occupancy model, etc.).
    - This will be reserved for studies that estimate shifts based on modeled estimates of occupancy or abundance
- *Temperature Variable Measured* (Column J)*:* Specific temperature variable measured in the study (eg., mean annual temperature, minimum in third quarter, etc.).
  - If temperature is not assessed, put “n/a”. See third tab for corresponding list of coded climate variables
  - **Note: Climate variables do not need to be directly measured in the study (i.e. authors can cite previous studies showing changes in these variables in the study area).**
- *Direction of Observed Change* (Column K)*:* Indicate how the temperature variable(s) changed (*increase, decrease, no change, or mixed).*
  - **Note: Select mixed if authors report changes in two or more temperature variables and the changes are not consistent.**
- *Precipitation Variable Measured* (Column L)*:* Specific precipitation variable measured in the study. Use the corresponding number from the “NEW Climate Variable Reference List” tab. If precipitation is not assessed, put “n/a”.
- *Direction of Observed Change (Precipitation)* (Column M)*:* Indicate direction of change in precipitation variable (*increase, decrease, no change, or mixed*).
  - **Note: Select mixed if authors report changes in two or more precipitation variables and the changes are not consistent.**
- *Author Assessment of Non-Climate Drivers* (Column N)*:* If assessed, indicate how the authors describe the role of non-climate drivers in explaining observed range shift.
- *Author Assessment of Sensitivity/Adaptive Capacity* (Column O)*:* If assessed, describe how the authors account for species sensitivity and/or adaptive capacity in explaining observed responses.
- *Study Area* (Column P)*:* Indicate study area as described by author. This should be the most specific description available (as low resolution as possible); we will use this to then create a shape file of the study area
  - If study includes multiple locations, list them in one cell, and do not separate into different rows. Indicate under Column F of the species level data tab which location each observation is from.
  - **Note: If authors report observations at multiple spatial scales for one study area (e.g. Paper 86), include only the largest spatial scale.**
- *Hemisphere* (Column Q)*:* Indicate which hemisphere the study occurs in (e.g. *northern, southern, both*)
- *Ecosystem* (Column R)*:* Indicate the general ecosystem (e.g. *terrestrial, marine, freshwater/aquati*c)
- *Habitat type* (Column S)*:* Indicate the type of habitats studied
- *Lat/Long 1-4* (Column T-AA)*:* Indicate the latitude and longitude coordinates if author provides
- *Relevant Fig* (Column AB)*:* Indicate the table, figure that contains a map or coordinates of the study area

Species-Level Data

- *Scientific Name* (Column AC)*:* Scientific name of focal species as provided in the original study. A new line in the spreadsheet should be established for each species analyzed in the paper**.**
  - **Note: If species is identified to subspecies level, enter third epithet (subspecies name) in column F**
- *Common Name* (Column AD)*:* Common name of focal species
- *Subspecies or Population* (Column AE)*:* If data in the paper is broken down according to any grouping *below* species, include the subgroup here.
  - This could be sex of the organism (i.e., range shifts data presented separately for males/females); age class (juvenile/adult); or geographic subpopulation (southern/northern population).
  - **Note: when relevant to a range shift hypothesis, a new line in the spreadsheet should be established for each subspecies/population.**
- *Dimension* (Column AF): Describe what kind of range shift the study attempted to assess: *latitude, longitude, elevation,* or *depth.*
- *Observation Type* (Column AG)*:* Describe whether the observation is of a shift in *occupancy*; or a shift in *abundance.* Coded as “obsvt” in SI figures & table.
  - *Occupancy shift*: a change in where the species is present or absent (e.g. shift in average latitude or maximum elevation)
  - *Abundance shift*: a change in the distribution of population (e.g. shift in optimum/maximum abundance or increase in abundance at the leading edge)
  - **Note: Measures of relative abundance should not be included, since we cannot determine whether increases are due to actual population growth or declines in comparison species.**
  - **Note: We cannot include measures of abundance (abundance increases or decreases) that occur in the range center- this doesn’t tell us anything about range shifts, only that a species became more abundant or less abundance within its range. Observations that are abundance, max/optimum, and categorized as abundance increase/decrease will be removed/excluded; observations that are abundance and are at an *edge* can be included.**
- *Parameter* (Column AH)*:* Describe what part or measure of the range the analysis applies to:
  - the *leading* edge of the range (poleward/upslope/deepest);
  - the *trailing* edge of the range (equatorial/downslope/shallowest);
  - the *mean* of the range (the middle latitude of a species’ range; often for “occupancy shifts” rather than abundance);
  - or the *maximum or optimum* (the point within the range that has the highest abundance and/or the mean of the range weighted by abundance; often for “abundance shifts”)
  - *East* or *West* of the range (for longitudinal shifts)
  - If multiple boundaries or measures are assessed, a new line in the spreadsheet should be established for each.
  - **Note: If the paper reports on part of a migratory species’ range, report whether it is the leading or trailing edge for just that portion of the range (e.g. if looking at the northern edge of the wintering range, put leading edge)**
- *Categorical Change* (Column AI)*:* Provide qualitative assessment of the observation:
  - *Latitudinal increase/decrease:* the boundary measurement (e.g., leading edge) increases or decreases in latitude.
    - This option can be used for abundance if the observation shows overall/mean/maximum abundance increasing or decreasing in latitude.
  - *Elevational increase/decrease:* the boundary measurement (e.g., leading edge) increases or decreases in elevation.
    - This option can be used for abundance if the observation shows overall/mean/maximum abundance increasing or decreasing in elevation.
  - *Abundance increase/decrease:* Abundance measures increased or decreased.
    - This option can be used for abundance observations at a single edge (e.g., where we don’t know whether there has been a latitudinal shift in abundance but there has been an increase or decrease in abundance at leading or trailing edge)
  - *Longitudinal shift*: a change in longitude (either east or west): west should be recorded as negative numbers, east is recorded as positive numbers
  - *Depth increase/decrease*: increase or decrease in depth.
  - *No change*: no change was observed or there was a change reported but it was not significant
    - **Note: Here, significance refers to whether the observed shift was found to be significantly different from 0.**
    - If papers only report significant findings (ie, if they study multiple species but explicitly state that they are not reporting non-significant findings), include all if possible (categorize non reported species as “no change”). If not possible (eg., non-significant species are not named), contact author or exclude if not available.
  - *Other:* If there was some other change, describe it in the Notes column (column AD)
  - If shifts reported at multiple spatial scales, record the largest scale only
- *Numeric Change* (Column AJ)*:* If available, describe the quantitative change
  - If shifts are reported quantitatively above the species level or qualitatively but there appears to be underlying quantitative data, indicate in comments of paper selection tab to contact authors and fill out a row in Data Contact spreadsheet
  - Additionally, if quantitative shifts are reported in a figure, but not a table (e.g. Dulvy et al 2008), enter the shifts qualitatively, but contact authors for exact numbers. If authors do not respond in a reasonable time frame, use [webplotdigitizer](https://automeris.io/WebPlotDigitizer/)
  - **Note: If authors report non-significant changes, enter the number that they report but categorize the observation categorically as “no change” in Column P**.
    - If authors only present results for species with significant changes, but it is clear that they assessed all species, then enter any species not reported as 0 for numeric change and “no change” for qualitative change.
  - Positive numbers indicate an increase in latitude (e.g., poleward movement); increase in elevation (e.g., upslope movement); and increase in depth (e.g., deeper movement). Negative numbers indicate equatorial movement, downslope movement, and shallower movement.
    - **Note: all eastern shifts should be recorded as positive; all western shifts should be recorded as negative (regardless of the positive/negative signs assigned in the paper)**
- *Metric/Unit* (Column AK)*:* Describe the associated metric (e.g., km/year, total meters change, degree latitude, etc.)
  - If using “degree”, be sure to indicate if it is latitudinal or longitudinal degree
- *Found in Table/Pg* (Column AL)*:* Where was the data reported? Give table, figure, or page number.
- *Conversion to km/decade* (Column AM)*:* Use numeric change and metric (columns Q & R) to convert quantitative change to km/decade equivalent.
  - Conversion from meters: x meters1000 *10# years of the study
  - Conversion from km:  x km *10# years of the study
  - Conversion from m/year: x meters/year1000* 10
  - Conversion from km/year: x km/year * 10
  - Conversion from degree latitude to km: x degrees latitude * 111km
    - When expressed as Degree, Minute, Second: 1 degree = 111km, 60 minutes in a degree (1 min = 1.85km), 60 seconds in a minute (1 sec = 0.03km)
    - When expressed as Decimal degrees: x latitudinal decimal degree * 111 (e.g., 1.7 decimal degree = 1.7*111km=188.7km)
- *Comparison to Historical Variability* (Column AN)*:* Was the observed change significant, based on historical variability in species range?
  - Select from *yes/no/not assessed*.
  - **Note: Only answer for studies that provide data about historical variability in species ranges and assess whether the observed shift was significant given that background variability. (e.g. Dulvy et al 2008 uses annual survey data, and calculates the depth anomaly from the 25 year mean). This is not the same as significance captured in column J. Here we are asking if the observed shift is significant compared to natural range shifts over time. Not all studies will test for this, but it is an ideal methodological component.**
- *Supports Temperature Hypothesis?* (Column AO): Assess qualitatively whether the observation supports, fails to support, or does not address our overarching temperature hypotheses.
  - Poleward, upslope, and deeper movements *support* temp hypotheses.
  - Equatorial, downslope, and shallower movements *fail to support*
  - If there is no change, select “*fails to support: no change*”
  - If the observed response was unclear, select “*unclear*”
  - If a temperature hypothesis was not assessed, select “*not assessed*”
  - **Note: Assess using our hypotheses (poleward, upslope, and deeper), not the authors**
- *Supports Precipitation Hypothesis* (Column AP): Assess qualitatively whether the observation supports, fails to support, or does not address our overarching precipitation hypotheses.
  - We hypothesize that the species will track its preferred precipitation niche or follow historical precipitation patterns
  - There is also a prevailing hypothesis that increased precipitation drives species downslope
  - **Note: Support will depend on precipitation change described in paper, as well as authors’ prediction of how species will move (i.e., we will rely on the authors to determine which direction of shift would follow their current niche).**
- *Significant Association with Climate Driver?* (Column AQ): If the study assesses whether the observed range shift was significantly associated with documented trend in climate driver, indicate here whether the relationship was significant.
  - *Yes/Not assessed*
- *Description of Climate Relationship* (Column AR): If assessed, indicate (qualitatively) how the authors describe the relationship between climate driver and range shift.
- *Notes* (Column AS): Any notes required to further understand previous columns; to provide context for the paper as a whole across multiple lines of the spreadsheet; or to note any unclear component of the paper.
- *Start Year* (Column AT)*:* The mid-point of the start year period of the study
- *End Year* (Column AU)*:* The mid-point of the end year of the study
- *Complete Taxonomic Information, Kingdom-Species* (Column AV-BB): Taxonomic information as imported from National Center for Biotechnology Information , Global Biodiversity Information Facility, Integrated Taxonomic Information System, Arctos, and/or iNaturalist databases.
- *Taxonomic Data Source* (Column BC): source of the taxonomic data in columns AT-AZ
- *Taxonomic Group* (Column BD)*:* Higher order taxonomic classification, used in range shift analysis.
