# Supplementary material for: Climate change and the global redistribution of biodiversity: substantial variation in empirical support for expected range shifts
Source: Environ Evid. 2023 Apr 11;12:7. doi: 10.1186/s13750-023-00296-0 (PMC11378804; doi:10.1186/s13750-023-00296-0)
Supplement: Supplementary file 5 — Additional file 5: Supplemental information on data analysis and results. Table S1. Sensitivity Analysis: Search protocol stopping criteria. Table S2. Overview of all models used to assess the direction and magnitude of shifts compared to general range shift expectations. Table S3. Significance of methodological variables in predicting magnitude of range shift. Table S4. Identifying & Evaluating Statistical Outliers. Table S5. Distribution of observations in the dataset. Table S6. Qualitative support vs fails to support. Table S7. Km/decade shifts by dimension (latitude, elevation, and depth) and parameter (leading edge, trailing edge, center-of-range). Table S8. Km/Dec Shifts by Taxonomic Grouping. Table S9. Relative Contribution of Ecological and Methodological Variables in Predicting Magnitude of Range shift. Table S10. Comparison of km/dec shift with previous meta-analyses. [file 13750_2023_296_MOESM5_ESM.docx]

**Additional file: Climate change and the global redistribution of biodiversity: are expected range shifts supported by the evidence?**

Madeleine A. Rubenstein, Sarah R. Weiskopf, Romain Bertrand, Shawn L. Carter, Lise Comte, Mitchell J. Eaton, Ciara Johnson, Jonathan Lenoir, Abigail J. Lynch, Brian W. Miller, Toni Lyn Morelli, Mari Rodriguez, Adam Terando, Laura M. Thompson

**Table S1. Sensitivity Analysis: Search protocol stopping criteria.**

We conducted a set of sensitivity analyses to validate the robustness of our search protocol (i.e., stopping criteria for the title/abstract search after reaching the asymptote of the accumulation curve). We searched 300 additional titles retrieved in the original search (selected at random), and evaluated whether their inclusion altered the findings of our analysis. We only found two studies which qualified for inclusion in the database. Neither study altered the magnitude of range shift assessment, since those papers did not provide quantitative range shift information (i.e., they only provided categorical descriptions of range shifts). For the direction of range shift assessment, the two additional studies led to the inclusion of 45 additional qualitative observations. None of these observations assessed precipitation-related range-shift hypotheses.

When we included the additional 45 observations of temperature-related range shifts, support for the temperature hypothesis did not change the overall conclusions of the qualitative analysis. The tables below compare the output of the logistic regression models for the original dataset (left columns, blue) and the sensitivity analysis dataset (right column, green). See SI Table 2 for full description of the models and study validity assessment section of main text for description of variables.

| **Dimension Model** | | | | | | |
| --- | --- | --- | --- | --- | --- | --- |
|  | Coefficient | | Standard Error | | Z value | |
| (Intercept) | -0.23 | -0.24 | 0.15 | 0.15 | -1.53 | -1.55 |
| Elevation (vs. depth) | 0.28 | 0.29 | 0.08 | 0.08 | 3.46 | 3.65 |
| Latitude (vs. depth) | 0.55 | 0.55 | 0.08 | 0.08 | 7.11 | 7.10 |
| Raw (vs. modeled) | 0.71 | 0.72 | 0.03 | 0.03 | 24.19 | 24.21 |
| Obsvt Occupancy (vs. abundance) | 0.21 | 0.22 | 0.03 | 0.03 | 6.50 | 6.72 |
| Sample NA (vs. irregular) | -0.41 | -0.42 | 0.04 | 0.04 | -11.52 | -11.68 |
| Sample Regular (vs. irregular) | 0.11 | 0.12 | 0.04 | 0.04 | 2.46 | 2.76 |
| ntax 2 (vs. 1) | -0.73 | -0.73 | 0.13 | 0.13 | -5.49 | -5.53 |
| Resurveyed (vs. Opportunistic | 0.15 | 0.15 | 0.03 | 0.03 | 4.85 | 4.77 |

| **Taxonomic Group Model** | | | | | | |
| --- | --- | --- | --- | --- | --- | --- |
|  | Coefficient | | Standard Error | | Z value | |
| (Intercept) | 0.26 | 0.26 | 0.17 | 0.17 | 1.57 | 1.55 |
| Bird (vs. Amphibian) | 0.12 | 0.11 | 0.11 | 0.11 | 1.12 | 1.06 |
| Crustacean (vs. Amphibian) | 0.17 | 0.16 | 0.15 | 0.15 | 1.10 | 1.03 |
| Fish (vs. Amphibian) | -0.17 | -0.18 | 0.11 | 0.11 | -1.49 | -1.58 |
| Insect (vs. Amphibian) | 0.56 | 0.55 | 0.11 | 0.11 | 5.28 | 5.19 |
| Mammal (vs. Amphibian) | -0.17 | -0.17 | 0.15 | 0.15 | -1.14 | -1.14 |
| Molluscs (vs. Amphibian) | 0.06 | 0.06 | 0.16 | 0.16 | 0.39 | 0.36 |
| Plant (vs. Amphibian) | 0.10 | 0.11 | 0.10 | 0.10 | 0.96 | 1.04 |
| Polychaetes (vs. Amphibian) | 1.45 | 1.44 | 0.21 | 0.21 | 7.02 | 6.98 |
| Reptile (vs. Amphibian) | -0.02 | -0.02 | 0.19 | 0.19 | -0.11 | -0.13 |
| Spider (vs. Amphibian) | 2.12 | 2.10 | 0.26 | 0.26 | 8.05 | 7.98 |
| Raw (vs. modeled) | 0.52 | 0.52 | 0.03 | 0.03 | 16.64 | 16.82 |
| Obsvt Occupancy (vs. abundance) | 0.19 | 0.20 | 0.03 | 0.03 | 6.02 | 6.25 |
| Sample NA (vs. irregular) | -0.66 | -0.65 | 0.04 | 0.04 | -18.05 | -17.90 |
| Sample Regular (vs. irregular) | -0.04 | -0.02 | 0.05 | 0.05 | -0.80 | -0.40 |
| ntax 2 (vs. 1) | -0.76 | -0.77 | 0.14 | 0.14 | -5.67 | -5.72 |
| Resurveyed (vs. Opportunistic | 0.18 | 0.17 | 0.03 | 0.03 | 5.57 | 5.56 |

|  | | | | | | |
| --- | --- | --- | --- | --- | --- | --- |
| **Parameter Model** | | | | | | |
|  | Coefficient | | Standard Error | | Z value | |
| (Intercept) | 0.37 | 0.38 | 0.14 | 0.14 | 2.71 | 2.73 |
| Trailing (vs. Leading) | -0.59 | -0.59 | 0.04 | 0.04 | -14.39 | -14.44 |
| Center-of-range (vs. Leading) | -0.02 | -0.02 | 0.03 | 0.03 | -0.54 | -0.60 |
| Raw (vs. modeled) | 0.67 | 0.67 | 0.03 | 0.03 | 21.44 | 21.48 |
| Obsvt Occupancy (vs. abundance) | 0.34 | 0.35 | 0.03 | 0.03 | 10.67 | 10.68 |
| Sample NA (vs. irregular) | -0.48 | -0.48 | 0.03 | 0.03 | -13.99 | -14.00 |
| Sample Regular (vs. irregular) | 0.09 | 0.10 | 0.04 | 0.04 | 2.10 | 2.22 |
| ntax 2 (vs. 1) | -0.88 | -0.88 | 0.14 | 0.14 | -6.49 | -6.51 |
| Resurveyed (vs. Opportunistic | 0.17 | 0.17 | 0.03 | 0.03 | 5.55 | 5.54 |

|  | | | | | |  |
| --- | --- | --- | --- | --- | --- | --- |
| **Ecosystem Type Model** | | | | | |  |
|  | Coefficient | | Standard Error | | Z value | |
| (Intercept) | 0.60 | 0.60 | 0.16 | 0.16 | -7.16 | 3.80 |
| Marine (vs. Freshwater) | -0.69 | -0.69 | 0.10 | 0.10 | 2.55 | -7.18 |
| Terrestrial (vs. Freshwater | -0.27 | -0.27 | 0.09 | 0.09 | -3.16 | -3.15 |
| Raw (vs. modeled) | 0.68 | 0.69 | 0.03 | 0.03 | 23.40 | 23.46 |
| Obsvt Occupancy (vs. abundance) | 0.28 | 0.28 | 0.03 | 0.03 | 9.18 | 9.22 |
| Sample NA (vs. irregular) | -0.55 | -0.55 | 0.03 | 0.03 | -16.21 | -16.22 |
| Sample Regular (vs. irregular) | 0.20 | 0.21 | 0.04 | 0.04 | 4.91 | 5.06 |
| ntax 2 (vs. 1) | -0.82 | -0.83 | 0.13 | 0.13 | -6.20 | -6.20 |
| Resurveyed (vs. Opportunistic | 0.14 | 0.13 | 0.03 | 0.03 | 4.37 | 4.36 |

**Table S2:** Overview of all models used to assess the direction and magnitude of shifts compared to general range shift expectations.

Methodological variables include: Data transformation (i.e., raw vs. modeled); Underlying nature of the data (i.e., abundance vs. occurrence); Monitoring Frequency (i.e., regular vs. irregular); Number of taxa (i.e., 1 vs. >1); Sampling design (i.e., resurveyed vs. opportunistic). See data validity section for full description.

| **Range Shift Direction** | | |
| --- | --- | --- |
| **Response variable** | **Fixed Effects** | **Random Effects** |
| Support vs. fail to support general range shift expectations | Dimension (latitude, elevation, depth)  Methodological variables | None |
| Support vs. fail to support general range shift expectations | Range shift parameter (leading edge, trailing edge, range-center)  Methodological variables | None |
| Support vs. fail to support general range shift expectations | Taxonomic group (taxonomic groups with >100 observations)  Methodological variables | None |
| Support vs. fail to support general range shift expectations | Ecosystem Type (freshwater, terrestrial, marine)  Methodological variables | None |
| **Range Shift Magnitude** | | |
| **Response variable** | **Fixed Effects** | **Random Effects** |
| Km/dec shift | Methodological variables | None |
| Estimated Marginal Means: Km/dec shift | Taxonomic group | Methodological factors |
| Linear Mixed Effects Model: Km/dec shift | Dimension, parameter, taxonomic group, and ecosystem type | Methodological factors |

**Table S3. Significance of methodological variables in predicting magnitude of range shift**

| **Methodological variable** | **Estimated range shift (km/dec)** | **SE** | **t-values** | **p-values** |
| --- | --- | --- | --- | --- |
| Single-taxa studies | 7.4315 | 3.151 | 2.358 | 0.0184 |
| Multi-taxa studies | -2.5124 | 0.577 | -4.354 | <0.05 |
| Raw (vs. modeled) | 4.37 | 0.47 | 9.24 | <0.05 |
| Resurveyed (vs opportunistic sampling) | -4.09 | 0.48 | -8.54 | <0.05 |
| Occupancy (vs abundance) | 3.48 | 0.49 | 7.12 | <0.05 |
| Sampling periods unclear (vs. irregular) | 3.57 | 0.55 | 6.51 | <0.05 |
| Sampling periods regular (vs irregular) | 14.49 | 0.64 | 22.49 | <0.05 |

This table reports the significance of methodological variables in predicting magnitude of range shift, as estimated by a multiple linear regression with km/dec as the response variable and all methodological variables as covariates. Estimated range shift coefficients (km/dec) should be interpreted relative to reference levels, rather than in absolute terms. The R2 for this model is 4.05%.

These findings suggest that studies which included multiple taxa have lower km/dec estimates than those which were single species studies, as did studies with resurveyed rather than opportunistic studies. Studies using measures of occupancy rather than abundance produced slightly higher estimates of km/dec shifts. Similarly, studies using raw, rather than modeled, data provided slightly higher km/dec shift estimates, as did studies with regular sampling as compared to irregular sampling periods. These findings are in line with previous meta-analyses which have identified similar relationships between methodological variables and estimated magnitude of range shifts (Brown et al 2016).

**Table S4. Identifying & Evaluating Statistical Outliers.** We assessed the sensitivity of our quantitative analysis to outliers. Although the average km/dec shifts estimated through our analysis of the overall database were similar to previous meta-analyses (Chen et al 2011, Lenoir et al 2020), there was substantial spread to the data (max=432.2, min=-305, sd=29.81). We therefore re-ran the quantitative analysis without outliers (defined as 1.5xinterquartile range) to evaluate the effect of including outliers on the overall estimates of range shifts. Excluding outliers did not change the overall conclusions for latitudinal and elevational shifts: species are shifting their ranges poleward and to higher elevations, and these shifts are significant (note that the estimated effect size is lower for both latitudinal and elevational shifts; Table 6). Excluding outliers from the depth observations, however, did alter the estimated effect size: rather than a non-significant shift to slightly shallower depths (-.0000924 km/dec, p=0.8679), we found a small but significant shift to deeper depths (0.000665, p=0.01073).

| Dimension | **Km/Dec shift** | min | max | sd | n | **p-value** | **Km/Dec shift (without outliers)** | min | max | sd | n | **p-value** |
| --- | --- | --- | --- | --- | --- | --- | --- | --- | --- | --- | --- | --- |
| latitude | **11.8** | -305.4 | 432.31 | 40.87 | 15790 | <0.05 | **6.876** | -39.87 | 59.8 | 18.45 | 11237 | <0.05 |
| elevation | **0.009** | -15.71 | 9.52 | 0.206 | 13333 | <0.05 | **0.00819** | -0.057 | 0.078 | 0.025 | 10519 | <0.05 |
| depth | **-.00009** | -0.12 | 0.084 | 0.013 | 739 | <0.05 | **0.000665** | -0.016 | 0.015 | 0.006 | 535 | <0.05 |

**Table S5. Distribution of observations in the dataset.** Distribution of observations throughout the range shift database. As in other meta-analyses (see SI Table 11), observations are biased towards latitudinal and elevational shifts; leading edge and center-of-range observations; Northern hemisphere observations; and observations in terrestrial ecosystems.

| **Element** | **N Observations** | **%** |
| --- | --- | --- |
| **Dimension** | |  |
| Elevation | 13347 | 40.90% |
| Depth | 739 | 2.26% |
| Latitude | 15795 | 48.40% |
| Longitude | 2751 | 8.43% |
| **Parameter** | |  |
| East | 561 | 1.72% |
| West | 507 | 1.56% |
| Leading edge | 12447 | 38.14% |
| Trailing edge | 3860 | 11.83% |
| Mean | 15257 | 46.75% |
| **Hemisphere** | |  |
| Northern | 29387 | 90.06% |
| Southern | 3110 | 9.53% |
| Both | 135 | 0.41% |
| **Ecosystem Type** | |  |
| Freshwater | 612 | 1.88% |
| Marine | 2820 | 8.64% |
| Terrestrial | 29200 | 89.48% |
| **Taxonomic Group** | |  |
| Amphibian | 528 | 1.62% |
| Ascidians tunicates and sea squirts | 2 | 0.01% |
| Bird | 7444 | 22.81% |
| Brittle stars | 16 | 0.05% |
| Centipedes | 21 | 0.06% |
| Crinoid | 1 | 0.00% |
| Crustacean | 380 | 1.16% |
| Fish | 2109 | 6.46% |
| Hydrozoa | 84 | 0.26% |
| Insect | 8813 | 27.01% |
| Mammal | 501 | 1.54% |
| Millipedes | 15 | 0.05% |
| Molluscs | 289 | 0.89% |
| Plant | 11734 | 35.96% |
| Polychaetes | 177 | 0.54% |
| Reptile | 232 | 0.71% |
| Sea anemones and corals | 26 | 0.08% |
| Sea cucumber | 2 | 0.01% |
| Sea urchin | 19 | 0.06% |
| Spider | 212 | 0.65% |
| Starfish | 27 | 0.08% |

**Table S6. Qualitative support vs fails to support**

**Direction of shift:** Overall support for shifts poleward, upward, and deeper across dimensions, parameters, taxonomic groups, and ecosystem types. We provide percentage support from the raw data (“Raw support”), as well as estimated marginal means (“EMM support”) and 95% confidence intervals (“EMM LCL” = lower confidence level; “EMM UCL” = upper confidence level) from our logistic regression models. For taxonomic groups, only groups with >100 observations and >1 paper were included in the logistic regression models.

| **Dimension** | **Number of Observations** | **Raw support** | **EMM support** | **EMM LCL** | **EMM UCL** |
| --- | --- | --- | --- | --- | --- |
| depth | 739 | 47.90% | 36.26% | 32.91% | 39.76% |
| elevation | 13335 | 37.41% | 42.85% | 41.79% | 43.92% |
| latitude | 15779 | 54.35% | 49.71% | 48.76% | 50.66% |

| **Parameter** | **Number of Observations** | **Raw support** | **EMM support** | **EMM LCL** | **EMM UCL** |
| --- | --- | --- | --- | --- | --- |
| leading edge | 12419 | 56.67% | 48.46% | 47.42% | 49.50% |
| trailing edge | 3848 | 35.00% | 34.07% | 32.50% | 35.69% |
| Center-of-range | 13586 | 40.73% | 47.91% | 46.90% | 48.93% |

| **Taxonomic Group** | **Number of Observations** | **Raw support** | **EMM support** | **EMM LCL** | **EMM UCL** |
| --- | --- | --- | --- | --- | --- |
| Amphibian | 416 | 445.09% | 40.82% | 36.20% | 45.62% |
| Ascidians tunicates and sea squirts | 2 | 0% | NA | NA | NA |
| Bird | 6070 | 42.62% | 43.65% | 42.34% | 44.98% |
| Brittle stars | 14 | 42.86% | NA | NA | NA |
| Centipedes | 21 | 85.71% | NA | NA | NA |
| Crinoid | 1 | 100.00% | NA | NA | NA |
| Crustacean | 321 | 55.76% | 44.90% | 39.41% | 50.51% |
| Fish | 1695 | 45.06% | 37.18% | 34.85% | 39.57% |
| Hydrozoa | 84 | 27.38% | NA | NA | NA |
| Insect | 8318 | 60.89% | 54.55% | 53.26% | 55.84% |
| Mammal | 417 | 36.21% | 36.81% | 32.17% | 41.71% |
| Millipedes | 15 | 93.33% | NA | NA | NA |
| Molluscs | 270 | 51.11% | 42.22% | 36.41% | 48.25% |
| Plant | 11570 | 37.52% | 43.09% | 41.96% | 44.23% |
| Polychaetes | 177 | 76.84% | 74.49% | 67.27% | 80.58% |
| Reptile | 178 | 43.26% | 40.21% | 33.16% | 47.69% |
| Sea anemones and corals | 26 | 42.31% | NA | NA | NA |
| Sea cucumber | 2 | 50.00% | NA | NA | NA |
| Sea urchin | 19 | 78.95% | NA | NA | NA |
| Spider | 212 | 91.04% | 85.14% | 78.08% | 90.22% |
| Starfish | 25 | 52.00% | NA | NA | NA |

| **Ecosystem Type** | **Number of Observations** | **Raw support** | **EMM support** | **EMM LCL** | **EMM UCL** |
| --- | --- | --- | --- | --- | --- |
| Freshwater | 596 | 54.19% | 53.77% | 49.61% | 57.89% |
| Marine | 2324 | 47.38% | 37.20% | 35.21% | 39.22% |
| Terrestrial | 26933 | 46.36% | 46.99% | 46.36% | 47.62% |

**Table S7. Km/decade shifts by dimension (latitude, elevation, and depth) and parameter (leading edge, trailing edge, center-of-range)**. Average shifts are reported in km/dec, and reflect raw (unmodeled) estimates of range shifts based on all observations in the database (i.e., across all parameters, ecosystem types, and taxonomic groups). P-values and 95% Confidence Intervals are derived from one-sample t-tests. Note: we did not include parameter-specific analysis for depth because of limited observations in this dimension.

| **Dimension** | **Km/Dec Shift** | **min** | **max** | **sd** | **n** | **p** | **95%CI (lower)** | **95%CI (upper)** |
| --- | --- | --- | --- | --- | --- | --- | --- | --- |
| Depth | 0.000 | -0.122 | 0.084 | 0.014 | 739 | 0.868 | -0.001 | 0.001 |
| Elevation | 0.009 | -15.714 | 9.524 | 0.206 | 13347 | 0.000 | 0.006 | 0.013 |
| Latitude | 11.805 | -305.400 | 432.316 | 40.872 | 15795 | 0.000 | 11.103 | 12.508 |

| **Parameter** | **Km/Dec Shift** | **min** | **max** | **sd** | **n** | **p** | **95%CI (lower)** | **95%CI (upper)** |
| --- | --- | --- | --- | --- | --- | --- | --- | --- |
| *Latitude* |  |  |  |  |  |  |  |  |
| Leading edge | 19.67 | -305.40 | 321.40 | 49.81 | 8248 | < 0.05 | 18.46 | 20.88 |
| Center-of-range | 4.19 | -189.25 | 432.32 | 25.75 | 6189 | < 0.05 | 3.53 | 4.85 |
| Trailing edge | 0.50 | -187.50 | 216.45 | 37.11 | 1358 | 0.75 | -2.57 | 3.56 |
| *Elevation* |  |  |  |  |  |  |  |  |
| leading | 0.01 | -15.71 | 9.52 | 0.38 | 4087 | 0.11 | 0.00 | 0.02 |
| Center-of-Range | 0.01 | -0.38 | 0.86 | 0.05 | 6884 | < 0.05 | 0.01 | 0.01 |
| trailing | 0.01 | -0.32 | 0.77 | 0.06 | 2376 | < 0.05 | 0.01 | 0.02 |

**Table S8. Km/Dec Shifts by Taxonomic Grouping.** Estimates of the magnitude of range shifts (expressed in km/decade) by taxonomic groups.

Estimates presented below are presented by dimension for latitudinal, elevational, and depth shifts using estimated marginal means (EMM; see methods section for fuller treatment of EMM). Modeled estimates of taxonomic-specific range shifts are presented only for taxonomic groups with 100 or more observations.

| **Latitude** | | | | | |
| --- | --- | --- | --- | --- | --- |
| **Taxonomic Group** | **Km/Dec shift (EMM)** | **SE** | **df** | **95% CI (lower)** | **95% CI (upper)** |
| Amphibian | -4.27 | 3.39 | 12894.00 | -15.67 | 7.14 |
| Bird | 9.68 | 0.87 | 12894.00 | 6.73 | 12.63 |
| Crustacean | 13.85 | 2.91 | 12894.00 | 4.04 | 23.66 |
| Fish | 11.43 | 1.49 | 12894.00 | 6.40 | 16.47 |
| Insect | 18.07 | 0.78 | 12894.00 | 15.43 | 20.71 |
| Mammal | 10.96 | 4.56 | 12894.00 | -4.38 | 26.31 |
| Molluscs | 16.22 | 3.15 | 12894.00 | 5.62 | 26.81 |
| Plant | -0.96 | 1.37 | 12894.00 | -5.56 | 3.64 |
| Polychaetes | 54.63 | 4.96 | 12894.00 | 37.93 | 71.33 |
| Reptile | -2.11 | 3.59 | 12894.00 | -14.19 | 9.97 |
| Spider | 67.65 | 2.82 | 12894.00 | 58.15 | 77.15 |
| **Elevation** | | | | | |
| **Taxonomic Group** | **Meter/Dec shift (EMM)** | **SE** | **df** | **95% CI (lower)** | **95% CI (upper)** |
| Amphibian | 36.94 | 14.31 | 11798.00 | -9.72 | 83.61 |
| Bird | 11.94 | 5.52 | 11798.00 | -6.07 | 29.95 |
| Crustacean | 17.86 | 210.36 | 11798.00 | -668.23 | 703.95 |
| Fish | -131.83 | 19.01 | 11798.00 | -193.84 | -69.82 |
| Insect | 27.68 | 5.98 | 11798.00 | 8.19 | 47.17 |
| Mammal | 5.16 | 12.72 | 11798.00 | -36.33 | 46.66 |
| Molluscs | -4.17 | 210.34 | 11798.00 | -690.22 | 681.88 |
| Plant | 6.98 | 2.45 | 11798.00 | -1.01 | 14.98 |
| Reptile | 14.42 | 28.20 | 11798.00 | -77.56 | 106.41 |
| Depth | | | | | |
| **Taxonomic Group** | **Meter/Dec shift (EMM)** | **SE** | **df** | **95% CI (lower)** | **95% CI (upper)** |
| Crustacean | -3.82 | 1.48 | 589.00 | -8.00 | 0.35 |
| Fish | 0.65 | 1.00 | 589.00 | -2.16 | 3.46 |
| Molluscs | -0.24 | 1.89 | 589.00 | -5.57 | 5.10 |
| Polychaetes | 0.77 | 2.09 | 589.00 | -5.11 | 6.66 |

**Table S9. Relative Contribution of Ecological and Methodological Variables in Predicting Magnitude of Range shift.**

Results from a linear mixed effects model to evaluate the relative contribution of ecological and methodological variables in explaining variance in the magnitude of range shifts (expressed as km/decade shifts). Methodological variables were set as random intercept terms; fixed effects included dimension, parameter, taxonomic classification, and ecosystem type.

Model structure: km/dec ~ 0 + dimension + parameter + taxonomic classification + ecosystem type + (1 | number of tax) + (1 | raw) + (1 | resurvey) + (1 | observation type) + (1 | sample regularity)

| **AIC** | **BIC** | **logLik** | **deviance** | **df.resid** |
| --- | --- | --- | --- | --- |
| 242242.3 | 242502.9 | -121089.1 | 242178.3 | 25403 |

| **Random effects:** |  | **Variance** | **Std Dev** |
| --- | --- | --- | --- |
| Groups | Name | Variance | Std.Dev. |
| sample | (Intercept) | 3.74 | 1.93 |
| obsvt | (Intercept) | 7.34 | 2.71 |
| resurvey | (Intercept) | 2.39 | 1.55 |
| raw | (Intercept) | 0.39 | 0.62 |
| ntax2 | (Intercept) | 8.74 | 2.96 |
| Residual | 798.44 | 28.26 |  |

| **Fixed Effects** | **Coefficient** | **Std. Error** | **df** | **t value** | **pvalue** |
| --- | --- | --- | --- | --- | --- |
| Dimension: depth | 5.12 | 4.87 | 11.02 | 1.05 | 0.32 |
| Dimension: elevation | 10.99 | 4.58 | 8.69 | 2.40 | 0.03 |
| Dimension: latitude | 20.99 | 4.60 | 8.87 | 4.56 | 0.00 |
| Parameter: mean | -7.40 | 0.56 | 5305.60 | -13.16 | < 2e-16 |
| Parameter: trailing edge | -6.18 | 0.71 | 17864.62 | -8.73 | < 2e-16 |
| Ascidians tunicates and sea squirts | -21.69 | 28.43 | 25428.68 | -0.76 | 0.45 |
| Bird | 1.65 | 1.58 | 22014.10 | 1.05 | 0.30 |
| Brittle stars | -16.10 | 8.13 | 25426.34 | -1.98 | 0.05 |
| Centipedes | 25.42 | 6.37 | 25383.38 | 3.99 | 0.00 |
| Crinoid | -16.91 | 28.43 | 25429.24 | -0.60 | 0.55 |
| Crustacean | -4.87 | 3.03 | 24562.76 | -1.60 | 0.11 |
| Fish | -7.59 | 2.97 | 24609.59 | -2.56 | 0.01 |
| Insect | 5.00 | 1.60 | 17167.38 | 3.13 | 0.00 |
| Mammal | 3.38 | 2.08 | 24743.29 | 1.63 | 0.10 |
| Millipedes | 33.35 | 7.47 | 25411.19 | 4.46 | 0.00 |
| Molluscs | -5.60 | 3.44 | 25310.97 | -1.63 | 0.10 |
| Plant | -0.82 | 1.49 | 25221.19 | -0.55 | 0.58 |
| Polychaetes | 7.80 | 3.78 | 24642.41 | 2.06 | 0.04 |
| Reptile | -6.01 | 2.60 | 25102.85 | -2.31 | 0.02 |
| Sea anemones and corals | -19.03 | 6.86 | 25431.26 | -2.77 | 0.01 |
| Sea cucumbers | -20.07 | 28.43 | 25429.24 | -0.71 | 0.48 |
| Sea urchin | -9.60 | 8.13 | 25421.43 | -1.18 | 0.24 |
| Spider | 55.64 | 2.54 | 22573.32 | 21.92 | < 2e-16 |
| Starfish | -16.71 | 6.51 | 25417.74 | -2.57 | 0.01 |
| Ecosystem Type: marine | 10.36 | 2.01 | 22850.95 | 5.14 | 0.00 |
| Ecosystem Type: terrestrial | -5.56 | 2.51 | 25296.29 | -2.21 | 0.03 |

**Table S10. Comparison of km/dec shift with previous meta-analyses**

| **Author** | **Year** | **Dimension** | **Parameter** | **Estimate** | **Notes** |
| --- | --- | --- | --- | --- | --- |
| Overall estimate: across dimension, parameters, & taxonomic groups | | | | | |
| This study | 2022 | All | All | 6.04 km/dec | Does not account for methodological factors |
| Parmesan and Yohe | 2003 | All | All | 6.1 km/dec | Does not account for methodological factors |
| Latitudinal shifts: all parameters & taxonomic groups | | | | | |
| This study | 2022 | Latitude | All | 11.81 | Does not account for methodological factors |
| Chen et al | 2011 | Latitude | All | 16.9 | Does not account for methodological factors |
| Latitudinal shifts: terrestrial | | | | | |
| This study | 2022 | Latitude | All | 11.12km/dec; significantly different from zero | Terrestrial only; accounts for methodology |
| Lenoir et al | 2020 | Latitude | All | 11.1 km/dec; not significant | Terrestrial only; accounts for methodology |
| Latitudinal shifts: marine | | | | | |
| This study | 2022 | Latitude | All | 19.71 km/dec | Marine only; accounts for methodology |
| Lenoir et al | 2020 | Latitude | All | 59 km/dec | Marine only; accounts for methodology |
| Pinsky et al | 2013 | [waiting for supplementary materials download] |  |  |  |
| Poloczanska et al | 2013 | Latitude | All | 30.6 km.dec | Does not account for methodological factors |
| Poloczanska et al | 2013 | Latitude | Leading edge | 72.0 km/dec | Does not account for methodological factors |
| This study | 2022 | Latitude | Leading edge | 24.58 km/dec | Does not account for methodological factors |
| Elevational Shifts: all parameters & taxonomic groups | | | | | |
| Rubenstein et al | 2022 | Elevation | All | 9.2 m/dec | Without accounting for methodology |
| Chen et al | 2011 | Elevation | All | 11.0 m/dec | Without accounting for methodology |
| Lenoir et al | 2020 | Elevation | Leading edge | 21.5m/dec | Terrestrial only ; accounts for methodological factors |
| This study | 2022 | Elevation | Leading edge | 13.6m/dec | Terrestrial only ; accounts for methodological factors |
| Lenoir et al | 2020 | Elevation | Trailing edge | 23.4m/dec | Terrestrial only ; accounts for methodological factors |
| This study | 2022 | Elevation | Trailing edge | 13.6 m/dec | Terrestrial only ; accounts for methodological factors |

**SI Table 10. Associated Citations**

Chen, I., Hill, J. K., Ohlemüller, R., Roy, D. B., & Thomas, C. D. (2010). *Rapid Range Shifts of Species of Climate Warming*. 49–52.

Lenoir, J., Bertrand, R., Comte, L., Bourgeaud, L., Hattab, T., Murienne, J., & Grenouillet, G. (2020). Species better track climate warming in the oceans than on land. *Nature Ecology and Evolution*, *4*(8), 1044–1059. https://doi.org/10.1038/s41559-020-1198-2

Parmesan, C., Yohe, G., Parmesan, C., Yohe, G., & Yohe, G. (2003). A globally coherent fingerprint of climate change impacts across natural systems. *Nature*, *421*(6918), 37–42. https://doi.org/10.1038/nature01286

Pinsky, M. L., Worm, B., Fogarty, M. J., Sarmiento, J. L., & Levin, S. A. (2013). Marine taxa track local climate velocities. *Science*, *341*(6151), 1239–1242.

Poloczanska, E. S., Brown, C. J., Sydeman, W. J., Kiessling, W., Schoeman, D. S., Moore, P. J., Brander, K., Bruno, J. F., Buckley, L. B., & Burrows, M. T. (2013). Global imprint of climate change on marine life. *Nature Climate Change*, *3*(10), 919–925.
