# Supplementary material for: Climate change and the global redistribution of biodiversity: substantial variation in empirical support for expected range shifts
Source: Environ Evid. 2023 Apr 11;12:7. doi: 10.1186/s13750-023-00296-0 (PMC11378804; doi:10.1186/s13750-023-00296-0)
Supplement: Supplementary file 6 — Additional file 6: ROSES systematic review checklist. [file 13750_2023_296_MOESM6_ESM.pdf]

Section/sub-section	Topic	Description	Further explanation	Checklist/meta-data	Author response	Comments
Title	Title	The title must indicate that it is a systematic review, and should indicate	The title should normally be the same or very similar to the review	Meta-data	Climate change and the	
Type of review	Type of review	Select one of the following types of review: systematic review,	See CEE Guidance on amendments and updates [1]	Meta-data	systematic review	
Authors' contacts	Authors' contacts	The full names, institutional addresses and email addresses for all		Checklist	Yes	
Abstract	Structured summary	The abstract of the manuscript must not exceed 500 words and must be		Checklist	Yes	
Background	Background	Describe the rationale for the review in the context of what is already	A theory of change and/or conceptual model should be presented that	Checklist	Yes	
Stakeholder engagement	Stakeholder engagement	The actual role of stakeholders throughout the review process (e.g. in		Checklist	Yes	
Objective of the review	Objective	Describe the primary question and secondary questions (when	The primary question is the main question of the review. The secondary	Checklist	Yes	
	Definition of the question components	Provide reference to the question key elements, e.g. population(s), intervention(s)/exposure(s), comparator(s), and outcome(s).	For other question types see [3,4]	Meta-data	Table 1 outlines all elements of the PECO framework for our study.	
Methods	Protocol	Provide citation, DOI or open-access link to published protocol.	The protocol should be peer-reviewed and publicly available online (open access).	Meta-data	Rubenstein, Madeleine A., et al. "Do empirical observations support commonly-held climate change range shift hypotheses? A systematic review protocol." Environmental Evidence 9.1 (2020): 1-10.	
	Deviations from protocol	Describe any ways in which the final methods of the review deviate from		Checklist	Yes	
Searches	Search strategy	Detail the search strategy used, including: database names accessed,		Checklist	Yes	
	Search string	Provide Boolean-style full search string and state the platform for which the string is formatted (e.g. Web of Science format)		Meta-data	TS=((“climate” OR “global 204 warming” OR "temperature" OR "precipitation") AND (“range” OR “distribution” OR “habitat 205 extent” OR “occupancy”) AND (“species”))	
	Languages - bibliographic	List languages used in bibliographic database searches		Meta-data	English	
	Languages – grey literature	List languages used in organisational website searches and web-based		Meta-data	English	
	Bibliographic databases	Provide the number of bibliographic databases searched		Meta-data	Included in our "search engine" section. We are using 2 bibliographic databse - Web of Science and Scopus	
	Web-based search engines	Provide the number of web-based search engines searched		Meta-data	Included in our "search engine" section. We are using 1 web based search engine - Google Scholar	
	Organisational websites	Provide the number of organisational websites searched		Meta-data	Not performed	
	Estimating comprehensiveness of	Describe the process by which the comprehensiveness of the search		Checklist	Yes	
	Search update	Describe any update to searches undertaken during the conduct of the	Compulsory (if update performed). A search update is good practice if	Checklist	Yes	
Article screening and study	Screening strategy	Describe the methodology for screening articles/studies for relevance.		Checklist	Yes	
	Inclusion criteria	Describe the inclusion criteria used to assess relevance of identified		Checklist	Yes	
Critical appraisal	Critical appraisal strategy	Describe here the method used for critical appraisal of study validity		Checklist	Yes	
	Critical appraisal used in synthesis	Describe how the information from critical appraisal was used in		Checklist	Yes	
Data extraction	Meta-data extraction and coding	Describe the method for meta-data extraction and coding for studies,	Optional, a map database can be included within a systematic review	Checklist	Yes	
	Data extraction strategy	Describe the method for extraction of qualitative and/or quantitative		Checklist	Yes	
	Approaches to missing data	Describe any process for obtaining and confirming missing or unclear		Checklist	Yes	
Potential effect modifiers/reasons	Potential effect modifiers/reasons	Provide a list of and justification for the effect modifiers/reasons for		Checklist	Yes	
Data synthesis and presentation	Type of synthesis	State the type of synthesis conducted as part of the systematic review (narrative only, narrative and quantitative, narrative and qualitative, narrative, qualitative and quantitative, narrative and mixed-methods)		Meta-data	Narrative, quantitative, and qualitative	
	Narrative synthesis strategy	Describe methods used for narratively synthesising the evidence base in		Checklist	Yes	
	Quantitative synthesis strategy	If data are appropriate for quantitative synthesis, describe methods for	Compulsory (if quantitative synthesis performed)	Checklist	Yes	
	Qualitative synthesis strategy	Describe methods used for synthesising qualitative data and justify your	Compulsory (if qualitative synthesis performed)	Checklist	Yes	
	Other synthesis strategies	Describe any other approaches used for synthesising data or combining	Compulsory (if other synthesis performed)	Checklist	n/a	
	Assessment of risk of publication	Describe methods for examining the possible influence of publication	This may be done for quantitative syntheses using diagnostic plots or	Checklist	Yes	
	Knowledge gap and cluster	Describe the methods used to identify and/or prioritise key knowledge	Optional	Checklist	n/a	
	Demonstrating procedural independence	Describe the role of systematic reviewers (who have also authored articles to be considered within the review) in decisions regarding inclusion or critical appraisal of their own work.	Reviewers who have authored articles to be considered within the review should be prevented from unduly influencing inclusion decisions, for example by delegating tasks appropriately.	Checklist	Yes	The subset of authors who are reviewing articles for inclusion do not have any published studies included in the review.
Results	Description of review process	Describe the review process including the volume of evidence identified		Checklist	Yes	
	Number of search results	Provide the number of search results from bibliographic databases	This number should not include web-based search engine or	Meta-data	94,672	
	Number of search results after duplicate removal	Provide the total number of search results from bibliographic database searches following duplicate removal.	This number should not include web-based search engine or organisational website searches: this will help assessment of the efficiency of the primary search string.	Meta-data	See methods section	
	Full text screening excludes	Additional file containing list of and reasons for full text exclusions.		Checklist	Yes	
	Title screening results	Provide the number of articles retained following title screening.	Optional if screening titles and abstracts together	Meta-data	12,098	
	Abstract screening results	Provide the number of articles retained following abstract screening.	Optional if screening titles and abstracts together	Meta-data	n/a	

	Title and abstract screening results	Provide the number of articles retained following title and abstract	Optional if screening titles and abstracts separately	Meta-data	n/a	
	Retrieval results	Provide the number of articles retrieved at full text.		Meta-data		842
	Unobtainable articles	Additional file containing list of unobtainable articles.		Checklist	Yes	
	Full text screening results	Provide the number of articles retained following full text screening.		Meta-data		315
	Consistency checking: screening	Results of consistency checking at all stages (screening, data extraction,		Checklist	Yes	
	Critical appraisal exclusions	If any studies are excluded due to low validity, provide the number of	Compulsory for any studies not included in synthesis due to validity.	Meta-data	n/a	
	Narrative synthesis	Describe the body of evidence identified using figures and tables,		Checklist	Yes	
	Extracted data	Additional file containing extracted quantitative or qualitative data		Checklist	Yes	
	Systematic map database	Additional file containing meta-data and coding for included studies.	Optional, a map database can be included within a systematic review	Checklist	Yes	
	Quantitative synthesis	Present results of quantitative synthesis of study findings (e.g. meta-	Compulsory (if quantitative synthesis performed)	Checklist	Yes	
	Qualitative synthesis	Present results of qualitative analysis of study findings (e.g. summaries	Compulsory (if qualitative synthesis performed)	Checklist	Yes	
	Other synthesis	Present results of any other synthesis methods used.	Compulsory (if other synthesis performed)	Checklist	Yes	
	Risk of publication bias	Describe the results of assessments for the possible influence of	For quantitative syntheses this may be done using diagnostic plots or	Checklist	Yes	
Discussion	Discussion	Discuss the review results and suggest further enquiry or analysis (e.g.		Checklist	Yes	
	Limitations of the review	Discuss possible limitations in the methods used.		Checklist	Yes	
	Limitations of the evidence base	Discuss possible limitations in the evidence base.		Checklist	Yes	
Conclusions	Implications for	Summarise the state of the evidence base and discuss the way in which	Reviews must not include practical environmental management	Checklist	Yes	
	Implications for research	Discuss the way in which the identified evidence may inform research	In this section some advocacy for future research on the reviewed topic	Checklist	Yes	
Declarations	Competing interests	Describe of any financial or non-financial competing interests that the		Checklist	Yes	

References

[1] Bayliss, H.R., Haddaway, N.R., Eales, J., Frampton, G.K. and James, K.L., 2016. Updating and amending systematic reviews and systematic maps in environmental management. Environmental Evidence, 5(1), p.20.

[2] Haddaway, N.R., Kohl, C., da Silva, N.R., Schiemann, J., Spök, A., Stewart, R., Sweet, J.B. and Wilhelm, R., 2017. A framework for stakeholder engagement during systematic reviews and maps in environmental management. *Environmental Evidence* , *6* (1), p.11.

[3] Collaboration for Environmental Evidence. 2018. Guidelines and Standards for Evidence synthesis in Environmental Management. Version 5.0. www.environmentalevidence.org/information-for-authors.

[4] Leeds Institute of Health Sciences. https://medhealth.leeds.ac.uk/info/639/information_specialists/1500/search_concept_tools. Accessed 12/11/2017.
